# A wolf in sheep's clothing? The interplay of perceived threat and social norms in hierarchy‐maintaining action tendencies towards disadvantaged groups

**DOI:** 10.1111/bjso.12849

**Published:** 2025-03-17

**Authors:** Nadine Knab, Melanie C. Steffens, Samer Halabi, Marie‐Therese Friehs, Arie Nadler, Boaz Hameri

**Affiliations:** ^1^ Department of Social, Environmental, and Economic Psychology, Faculty of Psychology University of Kaiserslautern‐Landau Landau Germany; ^2^ International Program for Conflict Resolution and Mediation, Department of Social Sciences Tel Aviv University Tel Aviv Israel; ^3^ Einstein Research Unit Coping with Affective Polarization, Center for Affective Neuroscience Charité – Universitätsmedizin Berlin Berlin Germany; ^4^ School of Psychological Sciences The Academic College of Tel Aviv‐Yafo Tel Aviv Israel; ^5^ Psychological Methods and Evaluation FernUniversität Hagen Hagen Germany; ^6^ School of Psychological Sciences Tel Aviv University Tel Aviv Israel; ^7^ The Evens Program in Conflict Resolution and Mediation Tel Aviv University Tel Aviv Israel

**Keywords:** dependency‐oriented helping, disadvantaged group, discrimination, helping behaviour, refugees, social inequality, social norms, threat

## Abstract

Almost inherently, helping occurs between people with disparate resources. Consequently, the helping dynamic can reinforce power hierarchies, particularly regarding dependency‐oriented helping (that preserves the power hierarchy) rather than autonomy‐oriented helping (that may level power hierarchies). We posit that perceived social norms regarding helping disadvantaged groups affect the tendencies to help versus discriminate. Specifically, individuals who feel threatened by disadvantaged groups may conform to social norms by offering dependency‐oriented help, thus preserving hierarchy while ostensibly adhering to societal expectations. Data from three correlational studies and one longitudinal study conducted in Germany (Studies 1a, 2a and 2b) and Israel (Study 1b) (combined *N* = 960) show that dependency‐oriented help towards refugees is higher when participants perceive strong norms to help but feel threatened at the same time. This interaction was not visible for autonomy‐oriented help. The finding is extended to a different intergroup setting (Study 3; *N* = 365) in which Jewish Israelis indicate higher intention to offer dependency‐oriented help to Arab Israelis when there is a high threat and strong norms perceptions (in contrast to weak norms). The results have theoretical and practical implications for understanding factors that influence hierarchy‐maintaining action tendencies and thereby intergroup inequality.

## INTRODUCTION

When the global number of refugees peaked at a historical high in 2015, reactions in destination countries were diverse. Overt hostility towards refugees emerged in various regions globally. For example, Israel, a neighbouring country to Syria, did not change their strict stance on asylum seekers when the numbers of refugees increased in 2015 (Hovil, 2015). In the United States, protests and political discussions surrounding refugees became increasingly polarized, showcasing a divided stance on the issue (Dreher et al., 2020). In 2017, Germany's political landscape saw the entry of the right‐wing party, AfD, known for its anti‐refugee stance, into the German Parliament as the third‐largest party, and it currently is the second strongest party in Germany (ZDF, 2023). This trend was not unique to Germany, as right‐wing movements gained traction in other countries, such as the rise of nationalist parties in Spain, Greece, Hungary and many more (Adler, 2023).

Despite the overt hostility, civil society played a pivotal role in meeting the newcomers' needs and helping them in various ways as politicians grappled with the challenges and struggled to respond swiftly to the increasing number of refugees in different parts of the world (e.g. Crepaz, 2020). For instance, in Canada, a country with a history of accepting refugees, communities organized welcome committees, provided support services and embraced newcomers (Canadian Council for Refugees, n.d.). Also, in Sweden, grassroots initiatives and volunteer efforts aimed at helping refugees gained momentum (but also opposite movements; Witte, 2015). In Germany, one of the European countries that received the highest number of refugees, a ‘Welcome Refugees’ culture developed with volunteers greeting refugees at train stations with flowers and teddy bears (Akrap, 2015). Hence, it appears that two antagonistic reactions to the higher number of refugees manifested globally. One was epitomized by the ‘welcome culture’ actively helping refugees – the other opposed refugees and sought to stem their influx. This research investigates whether these two reactions are in fact orthogonal and sheds light on more benign forms of maintaining power hierarchies.

The model of intergroup helping implies that a seemingly benevolent form of helping, termed dependency‐oriented helping (e.g., providing the full solution to a problem), places a disadvantaged group being offered help in a position of dependence and prevents them from taking control (Nadler, 2002). For example, in 2015, and continuously after (Schütze, & Elmar, 2023), politicians in Germany discussed handing out food stamps and coupons for refugees instead of giving the refugees the authority to decide for themselves what they want to do with the money they receive (WELT, 2015). Thus, part of the activities of the ‘welcome culture’ has the potential to perpetuate social inequality (Borderline‐Europe, 2018). We argue that both dependency‐oriented helping and overt discrimination can function as hierarchy‐maintaining actions taken by majority group members to keep minority group members in check. In the following, we outline how both types of hierarchy‐maintaining actions can be rooted in threat perceptions and argue that the presence of social norms to help a disadvantaged group may affect the degree of engaging in overt discrimination versus more covert discrimination like dependency‐oriented helping.

### Discrimination and helping as strategies to maintain intergroup hierarchy

Social identity theory postulates that people strive to maintain a positive image of their own group (Tajfel, 1982). In an intergroup context in which this positive ingroup image could be threatened by another group (an outgroup), ingroup members may engage in discrimination against that outgroup as a defence mechanism (Krosch, 2022). ‘Discrimination is usually treated in social psychology as negative, often aggressive behavior aimed at the target of prejudice or negative stereotype’ (Dolinski, 1996, p. 145). In contrast, helping behaviour is typically regarded as socially desirable. Indeed, plenty of psychological research has attempted to determine the predictors of helping between individuals and social groups with the aim of facilitating this type of behaviour (e.g., Schein, 2011; Steblay, 1987; Stürmer & Snyder, 2010). However, research suggests that taking a closer, more critical look at the drivers of helping behaviour could be warranted.

At its core, helping relations are symptomatic of an unequal distribution of resources, where the helper dispenses resources to those in need. According to Nadler (2002), intergroup helping can be used as a strategy to maintain power hierarchies between groups. More specifically, the model distinguishes between different types of helping. On the one hand, there is autonomy‐oriented helping, in which the helper provides the receiver with the tools required to solve a given problem on their own. On the other hand, there is dependency‐oriented helping, in which the helper solves the problem for the receiver. That is, dependency‐oriented help does not endow the receiver with the capability of dealing with the given problem on their own – rather, it creates and maintains dependence on the helper. A Chinese proverb illustrates the difference: ‘You give a poor man a fish and you feed him for a day. You teach him to fish and you give him an occupation that will feed him for a lifetime.’ In an asylum‐seeker context, the analogy is made that providing food and shelter may solve refugees' immediate problems, but for them to create an independent and self‐sustained life, they also need to be given the opportunity to develop skills and options to provide food and accommodation for themselves.

Although the negative consequences of discrimination are unambiguous, it is important to note that providing solely dependency‐oriented help may also have long‐term negative consequences for intergroup relations in at least three ways. First, as mentioned, dependency‐oriented help makes it exceedingly difficult for refugees to become fully integrated and equal members of society. Second, this form of help – seemingly benevolent – may actually advance and sustain stereotypical perceptions of refugees as lacking agency, ability and value, leading to further intergroup division and animosity (Nadler & Chernyak‐Hai, 2014). Third, the provision of dependency‐oriented help that is not needed nor requested may lead to the rejection of that help by the receiver, thus straining the helper‐receiver relationship and potentially obstructing future support or interaction (Nadler et al., 2009). These issues do not only pertain to refugee‐host relations but also to intergroup relations in general that are characterized by power imbalance. Thus, investigating factors that underpin more covert forms of maintaining social inequality, such as dependency‐oriented help, is necessary to understand what fosters and what hinders equal intergroup relationships.

### Threat

Intergroup threat occurs when one group believes another can harm them. In detail, concerns about physical harm or resource loss are termed realistic threats, while worries about the integrity of the ingroup's beliefs are termed symbolic threats (see Stephan et al., 2009). In addition, safety threats have been postulated to refer to worries regarding, for instance, public safety (Landmann et al., 2019). Past research has shown a positive association between threat perceptions and discrimination and the unwillingness to confront injustice (Celikkol et al., 2021; Krosch, 2022; Pereira et al., 2010). Nadler et al. (2009) found, somewhat counterintuitively, that if people feel that their group status in the social hierarchy is threatened by a lower‐status outgroup, they are more likely to provide help to that outgroup. Specifically, people perceiving status differences as unstable and changeable (and therefore threatening), were more likely to provide dependency‐oriented help to the status‐threatening group. The researchers explain this as a defensive act to thwart a threat to the ingroup's social identity. These results are in line with past research supporting the notion that perceived threat from a minority outgroup predicts the likelihood of the ingroup providing dependency‐oriented help rather than autonomy‐oriented help (Jackson & Esses, 2000). If threat predicts both overt discrimination and dependency‐oriented help (a more covert form of maintaining hierarchy), the question arises what moderates the likelihood of each of these behaviours being shown. We introduce social norms as a potential moderator impacting people's tendency to show dependency‐oriented help (and discrimination).

### Social norms

‘Social norms are customary standards or guides for behavior, attitudes and beliefs shared by a group’ (Murrar et al., 2020, p. 889), which can exert social pressure to perform or not perform a certain behaviour (Ajzen, 1991). Extensive research has investigated the question of exactly how norms influence behaviour (e.g., Cialdini et al., 1990; Kelman, 1961; Sherif, 1936; Tankard & Paluck, 2016). The influence of norms has been demonstrated in different contexts and used in various interventions, including for example, interventions to reduce private vehicle use (Kormos et al., 2015), increase energy conservation (Nolan et al., 2008) and facilitate prosocial behaviour (e.g., House, 2018; House et al., 2020). More pertinent to our research, past studies suggest that social norms may facilitate both helping behaviour and discrimination (Berkowitz, 1972; Sechrist & Stangor, 2001); however, this has never been investigated within a single study. The basic rationale is that when people perceive a norm to be helpful or positive towards a social group, they typically act accordingly. Consequently, whether outgroup discrimination is shown or not depends on the salient norms that exist in the given social context. Thus, in terms of host‐refugee relations or power relations in general, the prediction is that if norms that endorse helping the disadvantaged group are pervasive in a given social environment, then this should increase helping behaviour and reduce overt discrimination.

Taken together, research indicates that perceived threat and norms both have the potential to elicit consequential reactions in intergroup relations. The present research brings together these separate lines of past theorizing. Specifically, we investigate the impact on hierarchy‐maintaining behaviour in situations in which the effects of threat and norms come together: when people experience a certain degree of threat to their social group from a disadvantaged group that could jeopardize status relations, but at the same time perceive norms to help. We argue that in these cases, dependency‐oriented help promotes the need to maintain one's superiority while at the same time appearing to be benevolent and compliant with norms that endorse intergroup helping.

### Current research

**FIGURE 1:**
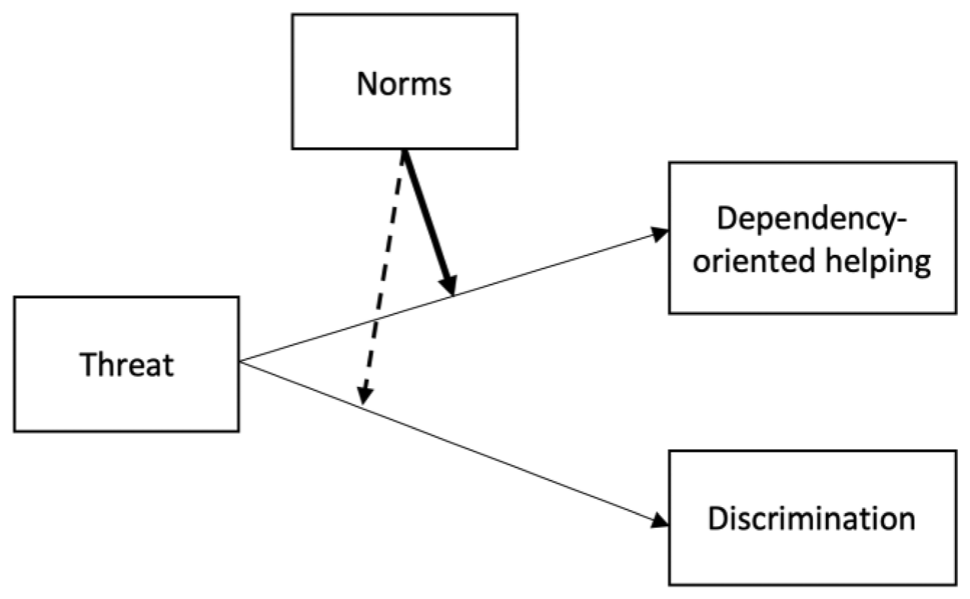
Theoretical scheme based on the presence of strong (bold line) or weak (dashed line) norms to help disadvantaged groups.

This research aims to clarify the role of perceived social norms in affecting responses to a perceived threat from a disadvantaged group, specifically focusing on how these norms influence the choice between overt discrimination and dependency‐oriented helping. Specifically, we tested the following main hypothesis: There is an interaction between perceived threat and social norms on dependency‐oriented helping. In detail, when perceived threat is high, the presence of strong norms to help disadvantaged groups is related to higher tendencies to provide dependency‐oriented help, as compared to when norms to help disadvantaged groups are weak. This relationship should not be visible for autonomy‐oriented help. Conversely, we hypothesize that strong social norms that endorse helping will reduce the likelihood of overt discrimination against disadvantaged groups, even among those who feel threatened. In essence, individuals may attempt to reconcile their fear of the disadvantaged group with the perceived normative pressure to help, leading them to offer help in a manner that preserves the social hierarchy (see Figure 1 for a depiction of threat and the associated action tendencies influenced by perceived norms).

In five studies, we investigate this question in various intergroup contexts and different countries. Studies 1a,b and 2a,b tackle the question in Germany and Israel regarding refugee‐host relations. Study 3 looks at a different intergroup setting: helping behaviour of Jewish Israelis to Arab Israelis. The studies received ethical approval from the respective institutions.

## STUDY 1A

The aim of Study 1a was to establish the connection of social norms and threat perceptions for the intention to offer dependency‐oriented help, autonomy‐oriented help and for agreement with discriminatory statements. The study was pre‐registered: https://aspredicted.org/V99_32K.

### Method

#### Participants

An a priori power analysis using the G*Power calculator (statistical test ‘linear multiple regression: fixed effects and *R* ^2^ increase’; Faul et al., 2009) indicated that a sample size of at least 353 participants was required for detecting a small effect of *f* ^2^ = 0.03 at a 5% level of significance and a power of 90%. We sampled 357 German participants, of which 127 identified as female, 227 as male and 3 as non‐binary (*M*_age_ = 39.90, SD = 12.07). Almost all participants had German nationality (98%); 7 people indicating other nationalities were removed from subsequent analyses, leaving 350 participants.

#### Procedure and measures

Participants were invited to a study on perceptions on refugees. The study took around 15 minutes and was administered by Clickworker – a data collection and crowd sourcing service located in Germany and the US – in February 2022. Participants were compensated with 2.20 Euros. Scales are described in the order in which they were administered. We collected additional variables intended for other research projects.^1^

##### Threat

We measured threat by using the scale proposed by Landmann et al. (2019). The scale differentiates between different types of threat. Realistic, symbolic and safety threat are associated closely with attitudes towards refugees, so we formed an overall scale termed direct threat, which was supported by a factorial analysis of the individual items (see OS).^2^ Each threat type was measured with three items. Examples for realistic threat, ‘The refugees living here threaten Germany's economic situation’, for safety threat, ‘The refugees living here threaten public safety in Germany’ and symbolic threat, ‘The refugees living here threaten our way of life and our values in Germany’ (*α* = .94). All items were displayed in a randomized order. Unless indicated otherwise all scales were from 1 (completely disagree) to 7 (completely agree).

##### Norms

We measured social norms by asking participants about their perception of 10 different social norms regarding helping refugees in their close social environment (i.e., family members and friends).^3^ Items were developed for the purposes of the current study (e.g., ‘Support for refugees is important’, *α* = .83). Weak norms conceptually included low support for refugees, but also actively not helping refugees (e.g., by support for deportations or giving money/support to ingroup‐members first). We introduced the items with: ‘In the following questions we are interested in what you think, that OTHERS think about the topic of refugees. Please indicate how many people in your social environment think that…’ On a 6‐point scale, answers ranged from 1 = almost no one to 6 = almost everyone (Paluck et al., 2016), with low scores indicating perceived weak social norms to help refugees.

##### Helping intentions

The different types of helping were measured by adapting the scale used in Becker et al. (2019). Dependency‐oriented helping was measured with four items,^4^ such as ‘I would sign a petition demanding that refugees are provided with everything they need to live through payments in kind (such as food coupons, clothes)’ (*α* = .67). Autonomy‐oriented helping was measured with 13 items such as ‘I would sign a petition demanding that refugees receive financial support from the government, so that they can provide for themselves’ (*α* = .94).

##### Discrimination

We measured agreement with discriminatory statements with 13 items, mainly operationalized by voicing preference for helping in‐group members (here Germans) or indicating no help at all, developed for the purposes of the current research (e.g., ‘I think it is more important to contribute to organizations that help Germans than to organizations that help refugees’; *α* = .95).

### Results and discussion

**FIGURE 2:**
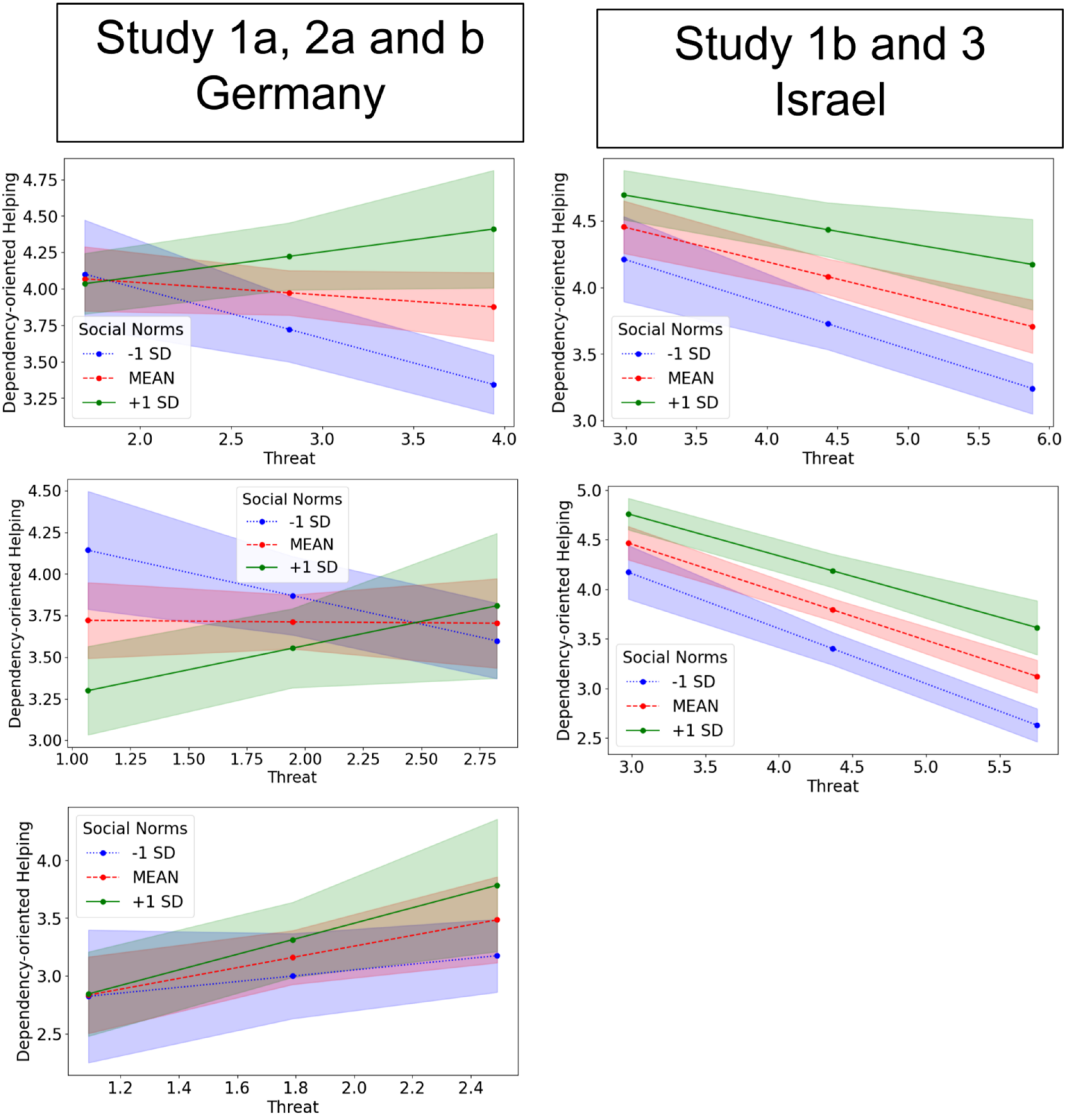
Interactions of norms and threat on dependency‐oriented helping in Studies 1a (upper left panel) and 1b (upper right panel), with findings in Germany presented on the left and findings in Israel, on the right. Full scales were 1–7 (dependency‐oriented helping) and 1–5 (threat). Shaded areas represent 95% CI.

Table 1 represents the mean values, standard deviations and correlations of the main variables. In all studies, we used multiple regression models with the PROCESS add‐on for SPSS (Hayes, 2018, Model 1 for moderation analyses) to analyse the interaction of social norms and threat perception on dependency‐oriented helping, autonomy‐oriented helping and discrimination (see Figure 2).

**TABLE 1:** Descriptive statistics and correlations for Study 1a.

	*M* (SD)	(1)	(2)	(3)	(4)
(1) Social norms	4.15 (1.05)				
(2) Direct threat	2.82 (1.21)	−.61**			
(3) Dependency‐oriented helping	3.80 (1.35)	.24**	−.21**		
(4) Autonomy‐oriented helping	3.50 (1.53)	.56**	−.64**	.46**	
(5) Discrimination	3.04 (1.53)	−.68**	.84**	−.14*	−.58**

*Note*: Threat on scale from 1 to 5, norms on scale from 1 to 6. All other variables on scales from 1 to 7.

*p < .05, **p < .01.

#### Dependency‐oriented helping

As hypothesized, results indicated a significant interaction of norms and threat on dependency‐oriented helping, *b* = 0.24, SE = 0.05, *t* = 4.45, *p* < .001, 95% confidence interval (CI) (0.13, 0.34), *R* ^2^‐change = .050. Specifically (see Figure 2, upper left panel), when social norms to help refugees in one's environment were perceived to be strong (i.e., mean + 1 SD), the provision of dependency‐oriented helping tended to increase the higher the threat perceptions (*b* = 0.17, SE = 0.10, *t* = 1.71, *p* = .088, CI [−0.03, 0.37]), offering weak support for our hypothesis. In contrast, when norms were perceived to be weak (i.e., one SD below the mean), then the provision of dependency‐oriented helping decreased significantly as threat increased (*b* = −0.33, SE = 0.09, *t* = 3.70, *p* < .001, CI [−0.51, −0.16]). Thus, there appears to be a shift in the direction of the relationship between threat perception and dependency‐oriented helping, contingent upon norm perceptions.

#### Autonomy‐oriented helping

Results indicate that the level of autonomy‐oriented helping, in contrast to dependency‐oriented helping, was not impacted by an interaction of norms and threat, *b* = −0.08, SE = 0.05, *t* = −1.60, *p* = .111, CI (−0.17, 0.02). Instead, there was a significant main effect of social norms on autonomy‐oriented helping, *b* = 0.59, SE = 0.15, *t* = −3.99, *p* < .001, CI (0.30, 0.88), such that higher perceived social norms were related to more autonomy‐oriented helping. Threat perceptions did not significantly impact the level of autonomy‐oriented helping but there was a tendency that the higher the threat, the less likely people agree to potentially provide autonomy‐oriented helping, *b* = −0.36, SE = 0.20, *t* = −1.79, *p* = .075, CI (−0.08, 0.04).

#### Discrimination

There was a significant interaction between norms in the social environment and threat predicting discrimination, *b* = −0.11, SE = 0.03, *t* = −3.22, *p* = .001, CI (−0.17, −0.04), *R* ^2^‐change = .008. Simple‐slopes tests showed that higher threat significantly predicted more discrimination when norms were perceived to be weak, *b* = 1.02, SE = 0.05, *t* = 18.87, *p* < .001, CI (0.92, 1.13). The same association was visible, but the relationship was weaker, when norms were perceived to be strong, *b* = 0.80, SE = 0.06, *t* = 13.03, *p* < .001, CI (0.68, 0.92).

#### Summary

We investigated if social norms and threat perceptions interact in predicting dependency‐oriented helping (but not autonomy‐oriented helping). Study 1a found support for this hypothesis. Thus, the provision of dependency‐oriented help seems to depend on perceived norms when threatened. Social norms and threat perceptions also interacted to predict agreement with discriminatory statements, which generally increased with higher threat, but less so if strong norms to help refugees were perceived. In short, when perceiving threat, strong norms to help refugees weakened the tendency to discriminate, while tending to increase dependency‐oriented helping.

## STUDY 1B

At the end of 2022, Israel had around 25,000 asylum seekers (UNHCR, 2023) – mostly from Sudan and Eritrea. The state of Israel denies the recognition and status as refugees (acceptance rate less than 1.5%; Peleg, 2022), which causes refugees' disadvantages regarding social security, access to health care and other social services (ASSAF, n.d.). Study 1b replicated Study 1a in a different country, preregistered here: https://aspredicted.org/42Z_BKN.

### Method

#### Participants

Based on the above a‐priori power analysis, we sampled *N* = 363 with the Israeli sampling company Midgam (participants were paid 5 NIS). Participants were Jewish Israelis; 187 indicated to be female, 178 male and 1 to not identify as either female or male. Age ranged from 17 to 74 (*M* = 41.87, SD = 16.48).

#### Procedure and measures

Participants received the same information as in Study 1a. We used the exact same measures, i.e., *threat* (*α* = .95), *perceived norms* (*α* = .83) *and discrimination* (*α* = .91), translated to Hebrew. We added two items to measure *helping intentions*, such that the final scale included 13 items to measure autonomy‐oriented helping (*α* = .94) and 5 items to measure dependency‐oriented helping (*α* = .83).^5^ Except for norms (scale 1–6) all scales were from 1 (completely disagree) to 7 (completely agree).

### Results and discussion

Table 2 represents the mean values, standard deviations and correlations of the main variables. As can be seen, perceived social norms to help refugees were lower than reported by German participants in Study 1a, and perceived threat was higher.

**TABLE 2:** Descriptive statistics and correlations for Study 1b.

	*M* (SD)	(1)	(2)	(3)	(4)
(1) Social norms	3.45 (0.86)				
(2) Direct threat	4.43 (1.45)	−.57**			
(3) Dependency‐oriented helping	4.01 (1.33)	.42**	−.43**		
(4) Autonomy‐oriented helping	3.11 (1.31)	.46**	−.63**	.75**	
(5) Discrimination	3.90 (1.23)	−.56**	.78**	−.47**	−.59**

*Note*: Norms on scale from 1 to 6. All other variables on scales from 1 to 7.

**p < .01.

#### Dependency‐oriented helping

Again, we found the expected significant interaction between norms in the social environment to help refugees and threat perceptions on dependency‐oriented helping, *b* = 0.09, SE = 0.04, *t* = 2.13, *p* = .034, CI (0.01, 0.18), *R* ^2^‐change = .01 (see Figure 2). Simple‐slopes tests showed that as expected, again, increasing threat significantly predicted less dependency‐oriented helping when norms were perceived to be weak, *b* = −0.34, SE = 0.06, *t* = −5.38, *p* < .001, CI (−0.46, −0.21). When social norms were perceived to be strong, this relationship was in the same direction this time, but much weaker, *b* = −0.18, SE = 0.06, *t* = −2.82, *p* = .005, CI (−0.31, −0.05).

#### Autonomy‐oriented helping

Again, the results indicated no interaction between threat and social norms, *b* = −0.01, SE = 0.04, *t* = −0.21, *p* = .834, CI (−0.08, 0.07). Higher threat perceptions were related to less autonomy‐oriented helping intentions, *b* = −0.46, SE = 0.14, *t* = −3.42, *p* = .001, CI (−0.73, −0.20). In contrast, social norms were not associated with autonomy‐oriented helping intentions, *b* = 0.27, SE = 0.17, *t* = 1.55, *p* = .123, CI (−0.07, 0.61).

#### Discrimination

The interaction of social norms and threat perceptions on discrimination replicated the pattern of Study 1a, *b* = −0.05, SE = 0.03, *t* = −1.95, *p* = .052, CI (−0.11, 0.00), albeit not significantly. Higher threat perceptions were related to more discrimination, *b* = 0.76, SE = 0.10, *t* = 7.60, *p* < .001, CI (0.56, 0.95). In contrast, social norms did not impact agreement with discriminatory statements, *b* = −0.04, SE = 0.13, *t* = −0.29, *p* = .885, CI (−0.29, 0.22).

#### Summary

Taken together, again, we found support for the hypothesis that the interplay of social norms and threat impacts the level of dependency‐oriented help, but not autonomy‐oriented help. Thus, high threat perception does not necessarily lead to no help at all, but under the condition of strong social norms could result in relatively higher levels of dependency‐oriented help. The results pattern differs, though, in interesting ways to the one in Germany. In Israel, dependency‐oriented helping generally decreased with higher threat – but strong social norms buffered the decline. Thus, strong norms led to higher levels of dependency‐helping than weak norms. Before discussing the differences in patterns, replications and extensions are reported. Furthermore, similar to the results in Germany, but only descriptively, the data collected in Israel showed that social norms could serve as a buffer for the agreement to discriminatory statements.

## STUDIES 2A,B

Studies 1a,b provided evidence for the interaction of social norms and threat perceptions on different action tendencies towards refugees. However, we did not specify the refugees' origin, which could affect perceived threat. It could be the case that stereotypes connected to refugees impact the assumed relationship of social norms and threat perceptions on hierarchy‐maintaining actions (see Fiske et al., 2002, for the relation between stereotypes and action tendencies). In Studies 2a,b, we used the context of Ukrainian refugees. After the start of the war between Russia and Ukraine, many refugees fled to other European countries. Despite the high number, media portrayed a less ambivalent reaction of host society members towards Ukrainian refugees. In particular, reactions appeared more positive, and politicians talked about two classes of refugees (see Wagner & Schwenken, 2023). We expected the same associations as in Studies 1a,b, albeit potentially weaker effects, as Ukrainian refugees could elicit less threat (Echterhoff et al., 2023; Sinclair et al., 2023). Pre‐registration: https://aspredicted.org/QCF_1HN.^6^

## STUDY 2A

Study 2a was implemented within a bigger research project that over the course of two months accompanied residents in a mid‐sized town in Germany who lived close to a new refugee shelter for people arriving from Ukraine. Participants were invited to partake in a study called ‘living together in (blinded for review)’ and received 14 Euros for their participation. Four measurement points were relevant (see Table 3). Our main goal was to replicate the findings from Study 1 in a naturalistic setting.^7^

**TABLE 3:** Measurement points of main variables in Study 2a.

Measurement 1	Measurement 2	Measurement 3	Measurement 4
Norms, threat	Dependency‐oriented helping, autonomy‐oriented helping, discrimination	Threat Ukrainian refugees (Subsample, *n* = 33)	Norms, threat, dependency‐oriented helping, autonomy‐oriented helping, discrimination

### Method

#### Participants

We recruited 110 German participants; 60 indicated to be female, 37 male and 13 did not identify as either female or male. Age ranged from 20 to 92 (*M* = 49.00, SD = 20.99).

#### Procedure and measures

Participants were recruited for a paper‐pencil survey, online survey or phone interview. At Measurement Point 1, threat perceptions did not specify the refugees' origin. Measurement Point 2 was one week later. A threat measure that specifically addressed Ukrainian refugees was included at Measurement Point 3 (three weeks later), as a preliminary test whether there are differential effects depending on the source of threat. Measurement Point 4 was nine weeks after Measurement Point 1 (see overview in Table 3). Due to time constraints, we reduced the number of items to three per scale. Unless indicated otherwise all scales were from 1 (completely disagree) to 7 (completely agree).

##### Threat

Direct threat was measured with three items, each pertaining to symbolic threat, realistic threat and safety threat, adapted from Landmann et al. (2019), for example ‘Refugees living here threaten our way of life and values in (city)’, *α*_1_ = .83, *α*_2_ = .84, and at Measurement Point 3, ‘Refugees from Ukraine threaten our German way of life and German values’, *α* = .73.

##### Norms

An example item for norms is, ‘How many people in (city name) support the uptake of refugees?’, *α*_1_ = .81, *α*_2_ = .85, scale from 1 (almost no‐one) to 5 (almost everyone).

##### Helping intentions

An example for dependency‐oriented helping is ‘I would sign a petition that demands that refugees are provided with everything they need to live through payments in kind (such as food coupons, clothes)’, *α* = .60, and for autonomy‐oriented helping ‘I would sign a petition that demands that refugees receive financial support from the government, so that they can provide for themselves’, *α* = .71.

##### Discrimination

An example item for discrimination was ‘I think it is more important to support organizations that support disadvantaged Germans than refugees’, *α* = .70.

### Results and summary

Table 4 shows descriptives and correlations.

**TABLE 4:** Mean values, standard deviations and correlations of main variables in Study 2a.

	*M* (SD)	1.	2.	3.	4.	5.	6.	7.	8.	9.	10.
1. Social norms 1	3.57 (1.04)										
2. Threat 1	1.99 (0.86)	−.49**									
3. Dependency‐oriented helping 1	3.58 (0.83)	−.10	−.09								
4. Autonomy‐oriented helping 1	3.32 (0.86)	.47**	−.51**	.06							
5. Discrimination 1	1.91 (0.88)	−.64**	.69**	−.03	−.54**						
6. Social norms 2	3.66 (1.04)	.71**	−.51**	.05	−.49**	−.58**					
7. Threat 2	1.91 (0.88)	−.46**	.85**	−.10	.41**	.64**	−.55**				
8. Dependency‐oriented helping 2	3.37 (0.92)	.05	−.04	.54**	−.08	−.04	.03	−.07			
9. Autonomy‐oriented helping 2	3.29 (0.91)	.36**	−.62**	.03	.74**	−.56**	.48**	−.58**	.18		
10. Discrimination 2	1.88 (0.83)	−.55**	.69**	.03	−.42**	.76**	−.65**	.68**	−.03	−.54**	
11. Threat Ukraine	1.56 (.66)	−.40**	.69**	−.13	−.47**	.52**	−.33	.74**	−.08	−.34	.72**

**p < .01.

#### Dependency‐oriented helping

We found again the hypothesized interaction between perceived social norms and threat on dependency‐oriented helping (measured one week later), *b* = 0.28, SE = 0.07, *t* = 3.93, *p* < .001, CI (0.14, 0.42), *R* ^2^‐change = .12. Mirroring the pattern from Study 1a (see Figure 2), more threat significantly predicted less dependency‐oriented helping when norms were perceived to be weak (*b* = −0.31, SE = 0.10, *t* = −2.98, *p* = .004, CI [−0.52, −0.10]); but not significantly and reversed when they were perceived to be strong (*b* = 0.29, SE = 0.16, *t* = 1.86, *p* = .065, [−0.02, 0.60]). When specifying the threat as resulting from Ukrainian refugees, a similar pattern resulted (see Figure 2) that was not statistically significant, *b* = 0.34, SE = 0.27, *t* = 1.27, *p* = .21, CI (−0.21, 0.88), *R* ^2^‐change: .05,^8^ presumably due to the small sample size (*N* = 33).

#### Autonomy‐oriented helping

As expected, such an interaction between threat and perceived social norms on autonomy‐oriented helping was not observed for the provision of autonomy‐oriented helping, *b* = 0.04, SE = 0.07, *t* = 0.66, *p* = .512, CI (−0.09, 0.18). Again, a main effect of threat perceptions showed that more threat led to less autonomy‐oriented helping, *b* = −0.05, SE = 0.23, *t* = −2.23, *p* = .028, CI (−0.95, −0.06), whereas there was no substantial impact of social norms on autonomy‐oriented helping, *b* = 0.14, SE = 0.15, *t* = 0.95, *p* = .343, CI (−0.15, 0.44).

#### Discrimination

Results indicated a tendency of an interaction between social norms and threat perceptions, *b* = −0.10, SE = 0.05, *t* = −1.97, *p* = .051, CI (−0.19, 0.00), in the same direction as in Studies 1a,b. There was no main effect of social norms on discrimination, *b* = −0.11, SE = 0.11, *t* = −1.04, *p* = 0.330, CI (−0.33, 0.10), but a main effect by threat perceptions: the higher the threat the more likely participants agreed with discriminatory statements, *b* = 0.76, SE = 0.17, *t* = 4.62, *p* < .001, CI (0.44, 1.09).

#### Regression paths across measurement points

**FIGURE 3:**
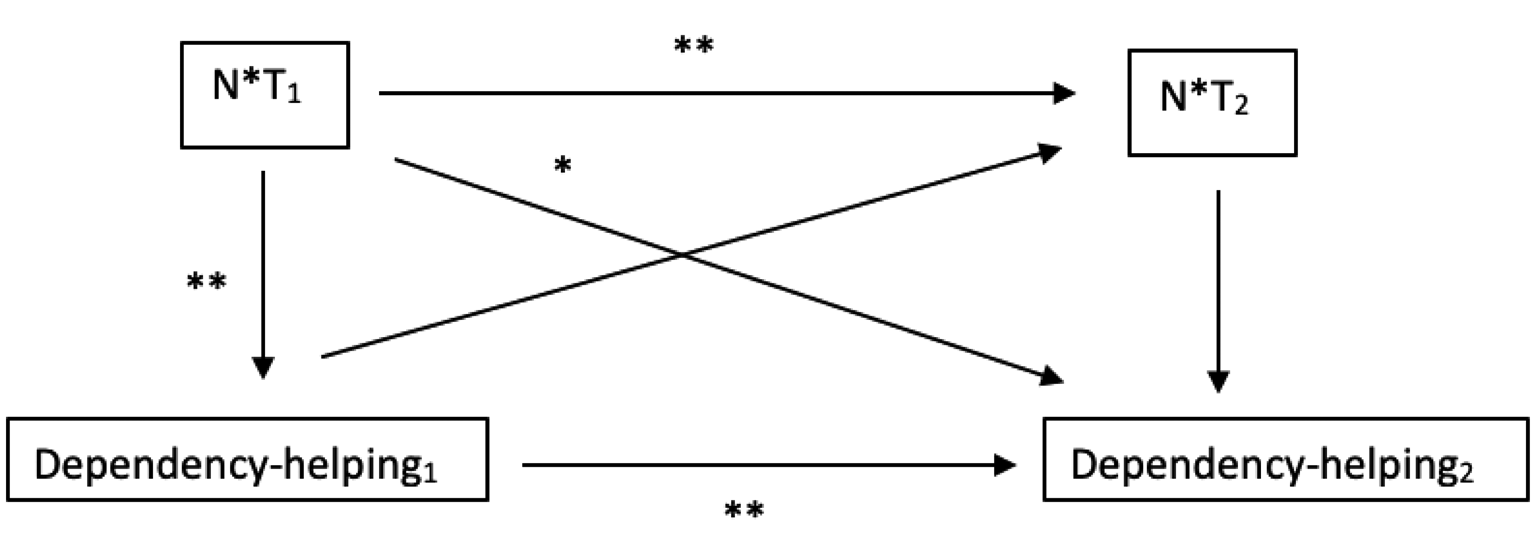
Cross‐lagged model of significant regression paths of interaction term between norms and threat (N*T, centered) and dependency‐oriented helping on their respective two measurement points in Study 2a. *p < .05, **p < .01.

Does the interaction of norms and threat at the first measurement point predict not only the intention for dependency‐helping measured one week later but also 8 weeks later? Results indicate that also eight weeks later, the provision of dependency‐oriented helping was impacted by the interaction of norms and threat perception at Measurement Point 1, *b* = 0.22, SE = 0.09, *t* = 2.35, *p* = .021, CI (0.03, 0.40). In contrast, testing the specificity of the relationship (see Figure 3), the first measure of dependency‐oriented helping did not significantly predict the interaction of norms and threat at the second measurement, *b* = 0.25, SE = 0.15, *t* = 1.68, *p* = .100, CI (−0.05, 0.54).

#### Summary

In Study 2a, we extended findings to a more naturalistic setting in a city in Germany with residents living close to a newly opened refugee shelter. We measured the dependent variables one week after the independent variables and also eight weeks after. Though only limited causal inferences are possible, the interaction of norms and threat perceptions impacted the level of dependency‐oriented helping (and not the other way around). Results indicate that strong social norms in the social environment led to a higher intention to provide dependency‐oriented help, when people simultaneously perceived a threat by refugees. A non‐significant, comparable pattern was present for threat perceived from Ukrainian refugees, possibly due to the sample size being too small for reliable conclusions. Consequently, in Study 2b, all variables were framed to tackle people's perceptions of Ukrainian refugees.

## STUDY 2B

In Study 2b we aimed to investigate the interplay of norms and threat on hierarchy‐maintaining action intentions in the context of Ukrainian refugees.

### Method

#### Participants, procedure and measures

Data were collected in Germany within a bigger project in which 130 participants answered the items specifically formulated towards Ukrainian refugees (55 identified as male, 74 as female, 3 as diverse, 1 as other), *M*_age_ = 41.62, SD = 12.93, range 20–80. Participants received either course credits or 5 Euros. We again used 3‐item measures for all scales: threat (symbolic, realistic and safety threat; *α* = .87), social norms (*α* = .85), dependency‐oriented helping (*α* = .66), autonomy‐oriented helping (*α* = .80) and discrimination (*α* = .75), anchored at 1 (completely disagree) and 7 (completely agree).

### Results and discussion

Table 5 shows descriptives and correlations.

**TABLE 5:** Descriptive statistics and correlations for Study 2b.

	*M* (SD)	(1)	(2)	(3)	(4)
(1) Social norms	3.89 (1.15)				
(2) Direct threat	1.82 (.74)	−.56**			
(3) Dependency‐oriented helping	3.05 (1.21)	.05	.12		
(4) Autonomy‐oriented helping	2.90 (1.13)	.51**	−.63**	.14	
(5) Discrimination	1.81 (.95)	−.54**	.58**	.21*	−.37**

*p < .05, **p < .01.

#### Dependency‐oriented helping

Specifying all items towards Ukrainian refugees shows a similar pattern to Study 1a and Study 2a, but not significant, *b* = 0.17, SE = 0.11, *t* = 1.57, *p* = .119, CI (−0.05, 0.39), *R* ^2^ change = .02. There were no significant main effects neither by threat nor by social norms, but descriptively, the typical pattern for German participants is observed: In the presences of strong perceived social norms to help refugees, more threat tends to go along with more dependency‐oriented helping.

#### Autonomy‐oriented helping

There was no significant interaction between threat and norms, *b* = −0.04, SE = 0.08, *t* = −0.54, *p* = .592, CI (−0.21, 0.12), nor a main effect by threat or social norms.

#### Discrimination

There was a significant interaction between norms and threat on the agreement with discriminatory statements, *b* = −0.21, SE = 0.07, *t* = −3.22, *p* = .002, CI (−0.34, −0.08). *R* ^2^ change = .04. Simple‐slopes tests showed again that threat significantly predicted discrimination when norms were perceived to be weak, *b* = 0.70, SE = 0.12, *t* = 6.07, *p* < .001, CI (0.47, 0.93); but not when they were perceived to be strong, *b* = 0.20, SE = 0.14, *t* = 1.42, *p* = .158, CI (−0.08, 0.49).

#### Summary

To summarize, in Study 2b, we aimed to test if the interaction effect generalizes also to refugees who are perceived as less threatening. All items specifically named Ukrainian refugees, who are usually helped more and perceived as less threatening in Europe (Sinclair et al., 2023; see also Xuereb, 2023). We found that, indeed, disadvantaged groups who are perceived as less threatening may not receive the exact same level of helping and discrimination, albeit the pattern seems comparable to the previous studies.

## STUDY 3

In sum, as Studies 2a,b showed, first, by adding a longitudinal design, we were able to investigate the relations between norms, threat and action tendencies over time. Second, by specifying the social group of refugees, it seems that the impact of norms and threat on hierarchy‐maintaining actions is generalizable, albeit effects are smaller for disadvantaged groups perceived as less threatening. To generalize the results to other intergroup contexts beyond refugee‐host relations, an additional study used the context of Israel with Arab Israelis as the disadvantaged group. According to the Bureau of Statistics, 21% of Israel's population in 2023 identifies as Arab Israeli (Central Bureau of Statistics, 2023), making it the biggest minority in the country. According to the Central Bureau of Statistics in Israel, there is tremendous socio‐economic inequality between Jewish Israelis and Arab Israelis, with half of the poor families in Israel being Arab Israeli (Khalaily et al., 2023). We investigated – depending on threat perceptions and norms to support Arab Israelis – the extent to which Jewish Israelis would support Arab Israelis in autonomy‐oriented or dependency‐oriented ways.

### Method

#### Participants

With the migdam panel, we recruited 409 Jewish Israelis between the age of 18 and 89 (*M* = 46.88, SD = 17.02), of which 193 identified as male and 216 as female.

#### Procedure and measures

Participants were invited to take part in ostensibly two different studies, a first study on opinion on current socio‐political situations and a second study on decision making processes related to donations on online platforms. They were informed that they will receive 6 NIS for their participation and participate in a lottery to potentially win another 100 NIS. First, participants indicated perceived threat from Arab Israelis, then answered the scale on social norms to help Arab Israelis (‘Study 1’). Then, they were asked to indicate their intentions on scales similar to the previous studies (‘Study 2’).^9^ Finally, we asked for subjective socio‐economic status, political orientation, demographics and debriefed participants.

##### Threat

We used three items per component of direct threat (i.e., realistic threat, symbolic threat and safety threat), similar to Study 1b, exchanging refugees with ‘Arab Israelis’ (*α* = .95).

##### Norms

Norms (9 items) were also measured with the same items as in Study 1a, adapted to context (*α* = .87).

##### Helping intentions

We measured dependency‐oriented helping with four items, such as ‘I would donate to organizations that focus on assisting Arab Israelis in need in receiving food and shelter’ (*α* = .83). A selection of four items from Study 1a was used to measure autonomy‐oriented helping, such as ‘I would donate money to an initiative that encourages Arab Israelis to exercise their rights in Israel’ (*α* = .88).

##### Discrimination

Also, a selection of four items were used to measure agreement with discriminatory statements such as ‘I think that donating to organizations that help underprivileged Jewish Israelis is more important than donating to organizations that help Arab Israelis in need’ (*α* = .76).

### Results and discussion

Table 6 shows mean values and intercorrelations.

**TABLE 6:** Mean values, standard deviations and correlations of main variables in Study 3.

	*M* (SD)	1.	2.	3.	4.
1. Threat	4.37 (1.39)				
2. Norms social environment	3.22 (1.00)	−.53**			
3. Autonomy‐oriented helping	3.12 (1.42)	−.65**	.54**		
4. Dependency‐oriented helping	3.74 (1.37)	−.63**	.54**	.80**	
5. Discrimination	4.23 (1.34)	.71**	−.54**	−.64**	−.60**

*Note*: Norms on scale from 1 to 6. All other variables on scales from 1 to 7.

#### Dependency‐oriented helping

We found the expected significant interaction of norms and threat on dependency‐oriented helping, *b* = 0.07, SE = 0.03, *t* = 2.30, *p* = .022, 95% CI (0.01, 0.13), *R* ^2^ = .01. Simple‐slopes tests showed that more threat went along with significantly less dependency‐oriented helping when norms were perceived to be weak (*b* = −0.55, SE = 0.06, *t* = −9.88, *p* < .001, CI [−0.66, −0.44]); when they were perceived to be strong, this relationship was weaker (*b* = −0.41, SE = 0.05, *t* = −7.78, *p* < .001, CI (−0.52, −0.31); see Figure 2).^10^

#### Autonomy‐oriented helping

Again, as expected, there was no interaction between norms and threat perceptions, *b* = −0.02, SE = 0.03, *t* = −0.76, *p* = .446, 95% CI (−0.08, 0.03), but instead, a main effect of threat perceptions, *b* = −0.44, SE = 0.11, *t* = −3.85, *p* < .001, CI (−0.66, −0.21), as well as of social norms, *b* = 0.48, SE = 0.15, *t* = 3.24, *p* = .001, CI (0.19, 0.77), with higher threat and weaker social norms going along with less autonomy‐oriented helping.

#### Discrimination

There was no interaction between norms and threat perceptions on discrimination, *b* = 0.00, SE = 0.03, *t* = 0.06, *p* = .954, 95% CI (−0.05, 0.06), but significant main effects of threat perceptions, *b* = 0.56, SE = 0.10, *t* = 5.55, *p* < .001, CI (0.36, 0.76), as well as of social norms, *b* = −0.31, SE = 0.13, *t* = −2.39, *p* = 0.018, CI (−0.57, −0.05), such that more threat and weaker social norms were related to higher discrimination intentions.

To summarize, in Study 3, we investigated the interplay between threat and norm perceptions for dependency‐oriented helping by Jewish Israelis towards Arab Israelis, thereby extending the evidence to another intergroup context characterized by social inequality. We found support for our hypothesis, such that when Jewish Israelis felt threatened by Arab Israelis, but perceived strong norms to help them, the intent to offer dependency‐oriented helping was higher than when there were weak norms. Interestingly, the result patterns for dependency‐helping shows similarities to Study 1b in Israel and differences from the studies conducted in Germany, which we discuss below.

### Aggregated data analysis

**FIGURE 4:**
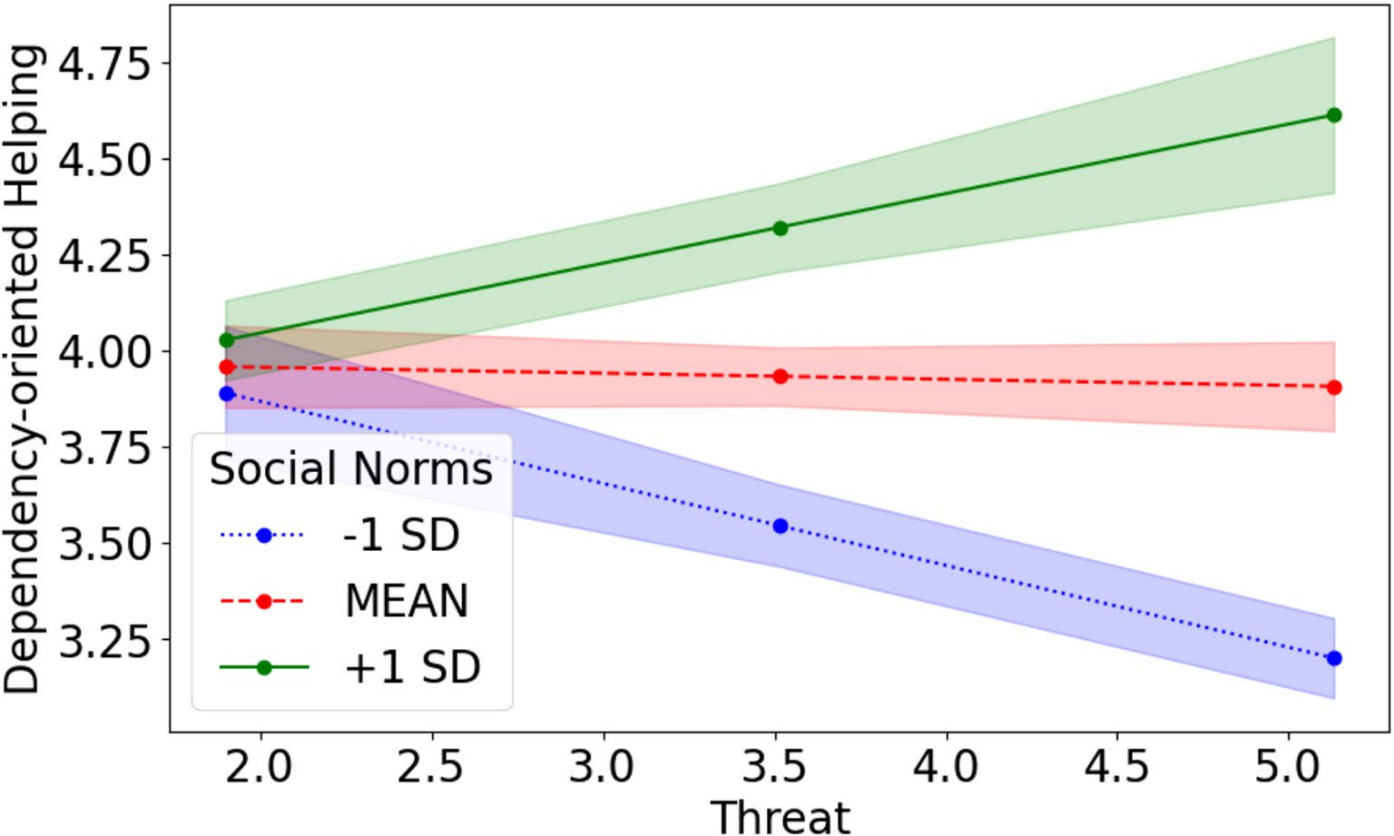
Interaction of norms and threat on dependency‐oriented helping across all studies. Full scales were 1–7 (dependency‐oriented helping) and 1–5 (threat). Shaded areas represent 95% CI.

To account for observed differences in patterns across the samples, particularly across countries, we aggregated the data and report the results including all subsamples (similar to Curran & Hussong, 2009).^11^ Combining all data (*N* = 1322) shows a significant interaction effect of social norms and perceived threat on dependency‐oriented helping, *b* = 0.18, SE = 0.02, *t* = 9.69, *p* < .001, CI (0.15, 0.22), *R* ^2^ = .06. Simple slopes analysis shows that the effect is especially strong for people indicating either weak social norms, *b* = −0.21, SE = 0.03, *t* = −7.14, *p* < .001, CI (0.15, 0.22) or strong social norms, *b* = 0.18, SE = 0.04, *t* = 5.11, *p* < .001, CI (0.11, 0.25), but not for people who perceive moderate social norms, *b* = −0.02, SE = 0.03, *t* = −0.62, *p* = .535, CI (−0.07, 0.03). For those with moderate perceptions of social norms, threat level had no effect on dependency‐oriented helping. In contrast, increased threat led to *less* dependency‐oriented helping among those perceiving weak social norms and *more* among those perceiving strong social norms (see Figure 4). There is no interaction effect on autonomy‐oriented helping, *b* = −0.01, SE = 0.02, *t* = −0.83, *p* = .406, (−0.05, 0.02). Thus, the effect is specific to dependency‐oriented helping also in the aggregated data analysis. Interestingly, the slight buffering effect of strong social norms when highly threatened on discrimination could not be observed in the aggregated data analysis, as the interaction between threat and perceived norms was not significant, *b* = 0.00, SE = 0.01, *t* = 0.09, *p* = .929, CI (−0.07, 0.03). Thus, irrespective of the level of social norms, discrimination rises when threat perception increases.

### General Discussion

Refugee movements and social inequality are on the rise (Auswärtiges Amt, 2024; United Nations, n.d.). An important question is which psychological factors influence how majority group members treat minority groups, often those at a disadvantage. This paper aims to explore the interplay between social norms and threat perceptions across different countries and intergroup contexts, focusing on their impact on various action tendencies. Specifically, we examined dependency‐oriented helping, a form of assistance that maintains, rather than alters, the power dynamics between disadvantaged and privileged groups. We thereby understand the (sole) offer of dependency‐oriented helping as another form of maintaining hierarchy and power relations. Our findings suggest that when individuals feel threatened by another social group, yet simultaneously perceive strong social norms encouraging them to help, they are more likely to engage in dependency‐oriented helping, as compared to a situation where perceived norms were weak. This pattern was not observed for autonomy‐oriented helping. Therefore, the interaction between threat perception and social norms appears to be particularly relevant to dependency‐oriented helping, and its mirror image was sometimes found for discrimination: highest agreement with discriminatory statements occurred in the presence of threat and weak social norms. However, the result on discrimination seems less robust, as the overall analysis did not yield a significant interaction effect on discrimination. Importantly, similar patterns of results were obtained across various intergroup contexts, demonstrating that the underlying mechanisms are not limited to refugee‐host relations.

The results of this study carry several theoretical implications. First, dependency‐oriented and autonomy‐oriented helping behaviours may be influenced by different factors. Throughout the studies, we observed no consistent effect of social norms on autonomy‐oriented helping. Although social norms did not consistently predict autonomy‐oriented helping, along with threat perceptions, they influenced agreement with discriminatory statements – a second hierarchy‐maintaining behaviour next to dependency‐oriented helping. In addition, dependency‐oriented helping and discrimination could both be conceptualized as ways of coping with perceived threat. When people feel threatened, they may respond by seeking to protect their sense of safety and identity (see e.g. McCrae, 1984). One way to cope with this threat could be through dependency‐oriented help, where the helper provides aid in a way that maintains the recipient's dependence, which could be motivated by a desire to reinforce the helper's own sense of control or superiority. This approach can create a dynamic where the helper remains in a dominant position, minimizing their own vulnerability. Alternatively, discrimination can also serve as a coping response, where individuals distance themselves from those they perceive as threatening by devaluing or excluding them. Discrimination would serve as a protective barrier, reinforcing in‐group cohesion and preserving a stable self‐concept. Although both strategies aim to manage feelings of insecurity, dependency‐oriented help superficially maintains relationships under unequal terms, whereas discrimination actively fractures social connections, distancing the helper from potential sources of threat. Future research could further explore these conceptual distinctions connected to coping and threat perceptions.

Our findings highlight the significance of conducting research across diverse cultural contexts. We chose Germany and Israel as suitable candidates because they represent distinct sociopolitical landscapes with differing levels of perceived threat and norms towards disadvantaged groups, offering a robust comparison for our hypotheses. Although in both Israel and Germany we demonstrated an interaction between social norms and threat perception regarding dependency‐oriented helping, the patterns were not identical. In Germany, dependency‐oriented helping increased with heightened threat and strong social norms (but not with weak norms). In contrast, in Israel, dependency‐oriented helping decreased as threat levels rose, though strong norms still promoted more of this type of helping.

One possible explanation for this difference could lie in the varying levels of perceived threat and social norms related to helping disadvantaged groups. In Israel, both disadvantaged groups (refugees and Arab Israelis) were perceived as highly threatening, accompanied by relatively low perceived norms for helping, unlike the situation in Germany. This heightened threat perception may reduce the intention to engage in dependency‐oriented helping, a form of aid that appears benign but maintains hierarchical relationships. It appears that the interaction we predicted was strongest when threat perceptions were, on average, rather low (below the scale midpoint in Germany, e.g., Study 1a), but not when they were, on average, rather high (above the scale midpoint in Israel, e.g., Study 1b). Future research could explore whether a high level of perceived threat reduces the influence of social norms on helping behaviour.

Moreover, dependency‐oriented helping was generally higher in the Israeli sample, potentially reflecting the communal solidarity prevalent in Israel, which may intensify during times of threat (World Jewish Congress, 2024). However, it remains uncertain whether this tendency extends to outgroup members (Van Hauwaert & Huber, 2020). Research by Ariely (2021) demonstrated that historical collective memory, in detail lessons derived from the holocaust, predicts attitudes and possibly actions towards refugees. Future studies could therefore explore the role of collective memories associated with historical transgressions in shaping intergroup relations and behaviours.

Despite the contributions of the studies, several limitations need to be mentioned. Throughout the studies, we encountered challenges with the measurement and distinction of dependency‐oriented and autonomy‐oriented helping. Even though the concepts have been increasingly used (e.g., Becker et al., 2019; Knab & Steffens, 2021; Nadler & Chernyak‐Hai, 2014; Wolf et al., 2024), a validated scale was not available when the research was conducted (but see Hanioti et al., 2024). For example, most items were designed in parallel, with the same action (e.g., signing a petition) applied to both dependency‐oriented and autonomy‐oriented helping, differing only in the cause. However, we cannot fully rule out the possibility that some items may have varied between the two types of helping in terms of cost and effort they require (which could also be the case in reality). Future research should aim to control for this factor more thoroughly.

In addition, our studies mainly include agreement to action intentions. Accumulating research has indicated that behaviour derived from behavioural intentions could have a smaller effect than the intentions imply (Webb & Sheeran, 2006). Nevertheless, more recent research proposes that this could depend on the specific intention‐behaviour link. Bø and Sjåstad (2024), for example, showed that participants were more moral than actually predicted or indicated. To better understand a potential intention‐behaviour gap, future studies could focus on concrete behaviours by collaborating with existing volunteers and organizations involved in charity and human rights efforts. Another option could be adapting economic games to the intergroup context differentiating various types of helping, for example, offering an option to change the rules modelling autonomy‐oriented support. Another valuable direction for future research could involve requiring participants to choose between distinct behavioural intentions (see Bareket et al., 2021), thus preventing them from both helping and discriminating simultaneously. This approach would strengthen the findings by clarifying underlying motivations, as participants would be limited to selecting only one option.

A further limitation is that almost all our studies were cross‐sectional, thus, evidence for causal relations is limited (but see OS for an experiment showing that participants in the threat condition, as compared to the control condition, were more likely to demonstrate dependency‐oriented helping intentions if they perceived strong norms to help refugees). The longitudinal design in Study 2a provides preliminary empirical evidence to rule out that the different types of helping or the agreement to discriminatory statements impact norm and threat perceptions. To exemplify, it is conceivable that expressing discrimination influences threat perceptions or even norms (e.g., Álvarez‐Benjumea, 2023).

In sum, in light of the prediction on increases rather than decreases in global refugee numbers (UNHCR, 2023), research on the determinants of inclusive versus divisive intergroup relations between host‐country members and refugees is timely and highly pertinent. One of the biggest tasks of the global community relates to successfully integrating refugees as equal members into the societies to which they flee. To achieve this, it may not suffice to donate clothes and food to refugee shelters (located often outside of cities). Rather, the host society needs to provide refugees with sufficient support and opportunities for personal growth and civic participation like autonomy, self‐representation, access to education and the job market. To mitigate these obstacles for the social integration of refugees and decreasing social inequality for disadvantaged groups in general, researchers should investigate the fundamental mechanisms that underpin these actions. In a society in which equal participation should be accessible to all, it is crucial for researchers and civil society to scrutinize seemingly benevolent actions, such as dependency‐oriented helping. These actions, while appearing helpful, may leverage on intergroup power relations and actually harm intergroup relations or even sow the seeds of conflict, acting as a wolf in sheep's clothing.

## AUTHOR CONTRIBUTIONS

**Nadine Knab:** Conceptualization; investigation; funding acquisition; writing – original draft; methodology; visualization; writing – review and editing; formal analysis; project administration; data curation; supervision; resources. **Melanie C. Steffens:** Conceptualization; supervision; writing – review and editing. **Samer Halabi:** Data curation; conceptualization; funding acquisition. **Marie‐Therese Friehs:** Data curation; funding acquisition. **Arie Nadler:** Supervision. **Boaz Hameri:** Supervision; writing – review and editing; conceptualization.

## CONFLICT OF INTEREST STATEMENT

The author(s) declare no potential conflicts of interest with respect to the research, authorship and/or publication of this article.

## Supporting information


Data S1.

## Data Availability

Data of all studies in this article are available on the Open Science Framework https://osf.io/a6uxq/?view_only=4c2256206f244168a2ffb155dbfeca79.

## Footnotes

^1^
For example: socio‐economic status and trait victimhood (Gabay et al., 2020).

^2^
Results of a respective factor analysis pertaining to the specific threats (separated by symbolic, realistic and safety threat) are reported in the Online Supplement (OS). Landmann et al. (2019) also measured extended threats such as overstrained institutions that have been connected more closely with general attitudes towards migration. These were included for research purposes unrelated to this manuscript.

^3^
We also measured perceived norms on a country‐level; results were comparable, but sometimes weaker, and are provided in the OS.

^4^
As we used mainly unvalidated scales, we conducted exploratory factor analysis to make sure we are able to distinguish between autonomy‐ and dependency‐oriented helping. Not all items for dependency‐oriented helping loaded on the factor as expected, so we selected only 4 items which did not overlap with the factor for autonomy‐oriented helping (see OS for more information).

^5^
The exploratory principal axis factor analysis for the helping items revealed similar items to fit as in Study 1, resulting in 5 items (including one item that was new in Study 1b).

^6^
Because of a last‐minute addition, the actual data collection comprised one additional measurement point.

^7^
In addition, we were interested in the way that proximity and contact potentially impact threat perceptions and thus the levels of dependency‐oriented helping. Unfortunately, contact experiences (positive and negative) did not change over the course of the study. Thus, we do not report the impact of number of contacts.

^8^
Results from social norms and dependency‐oriented helping at Measurement Point 2 as threat from Ukrainian refugees was measured after dependency‐oriented helping at Measurement Point 1. Using social norms at Measurement Point 1 increased the effect slightly: *b* = .49, SE = .34, *t* = 1.43, *p* = .164, CI (−.21, 1.18), *R* ^2^ = .06.

^9^
There was also another measurement of dependency‐oriented helping aiming to tap more into behaviour by charity‐giving. There were issues with the design (see OS), so we report the results only there.

^10^
Given the high correlation between dependency‐oriented helping and autonomy‐oriented helping (see Table 6), controlling for autonomy‐oriented helping slightly increased the effect size of the interaction, *b* = 0.09, SE = 0.02, *t* = 3.56, *p* < .001, CI (0.04, 0.13), *R* ^2^ = .01.

^11^
The syntax for the analysis is stored on the osf folder of the project.
