# Supplementary material for: A wolf in sheep's clothing? The interplay of perceived threat and social norms in hierarchy‐maintaining action tendencies towards disadvantaged groups
Source: Br J Soc Psychol. 2025 Mar 17;64(2):e12849. doi: 10.1111/bjso.12849 (PMC11912525; doi:10.1111/bjso.12849)
Supplement: Supplementary file 1 — Data S1. [file BJSO-64-0-s001.docx]

**A wolf in sheep’s clothing? The interplay of perceived threat and social norms in hierarchy-maintaining action tendencies towards disadvantaged groups**

**Online Supplemental Material**

[Study 1A/B 2](#_Toc178849916)

[Study 2A/B 10](#_Toc178849917)

[Study 3 11](#_Toc178849918)

[Experimental Study S1 14](#_Toc178849919)

[References OS 22](#_Toc178849920)

# Study 1A/B

**Factor Analysis helping intentions items (11 out of 13 from Becker et al., 2018, Data Study 1A)**

**Factor loadings marked with * are used for the analyses reported in main body of manuscript.**

*Table SM1*

Exploratory Factor Analyses Helping Items Study 1A.

|  | **Factor 1** | **Factor 2** |
| --- | --- | --- |
| **Autonomy 1:**  Ich würde eine Petition unterschreiben, die fordert, dass sich Flüchtlinge mithilfe einer finanziellen staatlichen Unterstützung vorrangig selbst versorgen können.  I would sign a petition that demands that refugees receive financial support from the government, so that they can provide for themselves. | .528* | .392 |
| **Autonomy 2:**  German: Ich würde Geld an eine Organisation spenden, die es geflüchteten Studierenden erleichtert, ihr Studium in Deutschland fortzusetzen.  I would donate money to an organization that makes it easier for refugee students to continue their studies in Germany. | .720* | .292 |
| **Autonomy 3:**  Ich würde Geld an eine Initiative spenden, die Flüchtlinge bezüglich ihrer Rechte in Deutschland bestärkt.  I would donate money to an initiative that encourages refugees to exercise their rights in Germany. | .858* | .210 |
| **Autonomy 4:**  Ich würde ehrenamtlich ein Projekt unterstützen, in dem Flüchtlinge und Bürger wöchentlich gemeinsam Ideen und Forderungen für die Flüchtlingspolitik entwickeln.  I would volunteer in a project where refugees and citizens meet weekly to collectively form ideas and demands for refugee policy. | .726* | .296 |
| **Autonomy 5:**  Ich würde Geld an eine Organisation spenden, die sich dafür einsetzt, dass Flüchtlinge auf dem Arbeitsmarkt gesetzlich gleichberechtigt sind  I would donate money to an organization that advocates that refugees have equal rights on the job market. | .827* | .209 |
| **Autonomy 6:**  Ich würde an einem Protest gegen das Arbeitsverbot von Flüchtlingen teilnehmen.  I would participate in a protest against the ban on work for refugees. | .606* | .217 |
| **Autonomy 7:**  Ich würde an einer Demonstration teilnehmen, um Abschiebungen von Flüchtlingen zu verhindern.  I would participate in a demonstration to prevent deportations of refugees. | .746* | .098 |
| **Autonomy 8:**  Ich würde eine Petition unterschreiben, dass Flüchtlinge sich frei im Land bewegen können.  I would sign a petition that advocates that refugees are able to move freely in Germany. | .692* | .176 |
| **Autonomy 9:**  Ich würde an einem Spendenabend teilnehmen, an dem für eine Organisation für kostenlose Rechtshilfe für Flüchtlinge gesammelt wird.  I would participate in a donation night where donations are collected for free legal aid to refugees. | .856* | .175 |
| **Autonomy 10:**  Ich würde eine Petition unterschreiben, die sich dafür einsetzt, dass Studienabschlüsse von Flüchtlingen anerkannt werden.  I would sign a petition that supports the acknowledgement of refugees’ degrees | .563* | .250 |
| **Autonomy 11:**  Ich würde eine Petition unterschreiben, die fordert, dass Flüchtlinge die schon einen Führerschein haben, nicht nochmal eine Prüfung ablegen müssen.  I would sign a petition that demands that refugees, who already have a driver’s permit, do not have to go through another driving test. | .514* | .218 |
| **Autonomy 12:**  Ich würde Inhalte auf Facebook und sozialen Medien teilen, die sich für die Aufnahme und Integration von Geflüchteten aussprechen.  I would share content on Facebook and other social media, that supports the uptake of refugees. | .735* | .185 |
| **Autonomy 13:**  Ich würde mich aktiv dafür einsetzen, dass Geflüchtete mehr Chancen in unserer Gesellschaft haben (z.B. durch die Kontaktaufnahme mit lokalen Politiker/innen).  I would activeley engage in activities that would support refugees‘ options to be part of our society (e.g. by contacting local politicians). | .738* | .317 |
| **Dependency 1: ***  Ich würde eine Petition unterschreiben, die fordert, dass Flüchtlinge durch Sachleistungen (z.B. Essensgutscheine, Kleidung) mit allem versorgt werden, was sie zum Leben brauchen.  I would sign a petition that demands that refugees are provided with everything they need to live through payments in kind (such as food coupons, clothes). | .071 | .535* |
| **Dependency 2:**  Ich würde Geld an eine Organisation spenden, die Vormünder für erwachsene Flüchtlinge vermittelt, die sich um deren amtliche Angelegenheiten kümmern.  I would donate money to an organization that arranges guardians for adult refugees, who take care of their legal issues. | .613 | .425 |
| **Dependency 3:**  Ich würde Geld an eine Initiative spenden, in der Ehrenamtliche abends vor Flüchtlingsunterkünften heißen Tee und frisches Obst an Flüchtlinge verteilen.  I would donate money to an initiative, in which volunteers hand out hot tea and fresh fruit to refugees in front of refugee accommodation centers in the evenings. | .743 | .276 |
| **Dependency 4:**  Ich würde ehrenamtlich ein Projekt wie die Tafel unterstützen, bei der gegen Vorlage eines Ausweises wöchentlich Nahrungsmittel an Flüchtlinge ausgegeben werden.  I would volunteer in a project like a soup kitchen, where upon production of an ID card, food is given weekly to refugees. | .654 | .327 |
| **Dependency 5:**  Ich würde Geld an eine Organisation spenden, die sich dafür einsetzt, dass hochqualifizierte Flüchtlinge wenigstens als PraktikantInnen oder 1?-Jobber arbeiten können.  I would donate money to an organization that advocates that highly qualified refugees can at least work as interns or 1€-workers. | .412 | .506 |
| **Dependency 6:**  Ich würde an einem Protest einer Initiative teilnehmen, die sich dafür einsetzt, dass allen Fl�chtlingen deutsches Betreuungspersonal zugewiesen wird.  I would participate in a protest by an initiative that advocates that all refugees are assigned German guardians. | .524 | .420 |
| **Dependency 7:**  Ich würde an einer Demonstration teilnehmen, in der sich Personen für direkte Nachbarschaftshilfe einsetzen, wie beispielsweise unaufgefordert Pfandflaschen an Flüchtlinge zu spenden.  I would participate in a demonstration where people speak out for direct neighborhood help such as the unsolicited donation of returnable bottles to refugees. | .673 | .293 |
| **Dependency 8:**  Ich würde eine Petition unterschreiben, die sich für ein Belohnungssystem für Flüchtlinge einsetzt, das je nach erreichtem Sprachniveau finanzielle Belohnungen vorsieht.  I would sign a petition that advocates for a reward system for refugees that gives financial rewards depending on the achieved language level. | .309 | .529* |
| **Dependency 9:**  Ich würde an einem Spendenabend teilnehmen, an dem Nachbarn spenden und Flüchtlinge Gerichte aus ihrem Heimatland kochen.  I would participate in a donation night where neighbors donate and refugees cook meals from their home countries. | .687 | .313 |
| **Dependency 10:**  Ich würde eine Petition unterschreiben, die Flüchtlingen einen Bewährungszeitraum zusichert, in dem sie die Chance haben ihren Integrationswillen zu zeigen.  I would sign a petition that ensures a probationary period to refugees where they are given a chance to show their will to integrate. | .100 | .648* |
| **Dependency 11:**  Ich würde eine Petition unterschreiben, die fordert, dass auch noch nach erfolgreichem Asylverfahren, Flüchtlinge Vormünder gestellt bekommen, die ihnen bei der Integration helfen sollen.  I would sign a petition that demands that even after a successful asylum proceeding, refugees are assigned guardians that help them to integrate | .193 | .545* |

Extraction Method: Principal Axis Factoring.

Rotation Method: Varimax with Kaiser Normalization.

*Table SM2*

Exploratory Factor Analyses Threat Items Study 1A. Based on eigenvalue > 1, one factor was retained. The factor had an eigenvalue of 6.74, explaining 74.84 % of the variance.

|  | **Factor 1** |
| --- | --- |
| **Symbolic threat 1:**  Die Einwanderung durch Flüchtlinge beeinträchtigt die deutsche Kultur. | .854 |
| **Symbolic threat 2:**  Die Werte und Überzeugungen der Flüchtlinge sind mit denen der Deutschen unvereinbar. | .799 |
| **Symbolic threat 3:**  Flüchtlinge, die hier leben, bedrohen unsere Lebensweise und unsere Werte in Deutschland. | .883 |
| **Realistic threat 1:**  Die Kosten für Integrationsprogramme für Flüchtlinge belasten Deutschland. | .799 |
| **Realistic threat 2:**  Die Einwanderung durch Flüchtlinge erhöht die Steuerbelastung für Deutsche. | .814 |
| **Realistic threat 3:**  Flüchtlinge, die hier leben, bedrohen die wirtschaftliche Lage in Deutschland. | .839 |
| **Safety threat 1:**  Die Flüchtlinge, die hier leben, bedrohen die Sicherheit in Deutschland. | .919 |
| **Safety threat 2:**  Durch den Zuzug von Flüchtlingen gibt es mehr Straftaten in Deutschland. | .799 |
| **Safety threat 3:**  Der Zuzug von Flüchtlingen macht Überfälle und Diebstahl wahrscheinlicher. | .883 |

Extraction Method: Principal Axis Factoring.

Rotation Method: Varimax with Kaiser Normalization.

**List of items social norms in close social environment (English/Hebrew)**

How many people in your close social environment (family, friends):


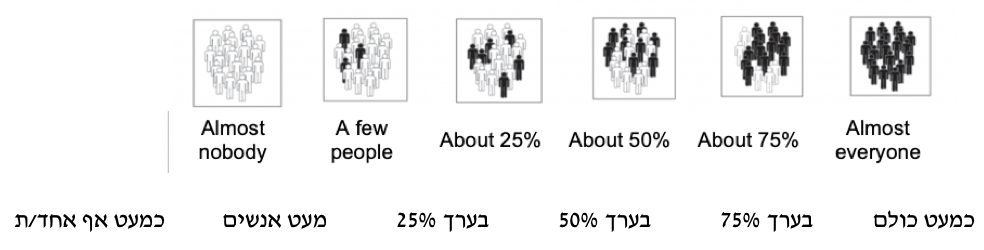


| …support the uptake of refugees | תומכים בקליטת פליטים |
| --- | --- |
| …want to help refugees | רוצים לעזור לפליטים |
| …see it as a moral obligation to help refugees | תופסים את העזרה לפליטים כחובה מוסרית |
| …dislike refugees, just because they are refugees | לא אוהבים פליטים רק בגלל שהם פליטים |
| …think /Israelis should be helped first, before refugees are helped. (rev.) | חושבים שצריך קודם לעזור לישראלים לפני שעוזרים לפליטים |
| …show hostility towards refugees | מפגינים עוינות כלפי פליטים |
| …think that refugees should be expelled from the country | חושבים שצריך לגרש פליטים מהמדינה |
| …think under certain circumstances it is acceptable to behave aggressively towards refugees | חושבים שתחת נסיבות מסוימות זה בסדר להתנהג בצורה אגרסיבית כלפי פליטים |
| …think options of assistance for refugees are good | חושבים שהאפשרויות סיוע לפליטים הן טובות |
| …speak out in favor of help for refugees | מדברים בעד עזרה לפליטים |

**Results of social norms on perceived country/city level and threat on dependent measures.**

**Study 1A:**

*Dependency-oriented helping*: *b* = .27, *SE* = .09, *t* = 3.04, *p* =.003, *CI* {.10;.44}, R^2^-change .024, main effects: threat, *b* = -1.22, *SE* =.34, *t* =-3.56, *p* <.001, CI {-1.90; -.55}, norms country-level: *b* = -.55, *SE* = .28, *t* =-1.95, *p* = .052, CI {-1.11;.01}

*Discrimination*: *b* = -.10, *SE* =.05, *t* = -1.84, *p* =.067, *CI* {-.21;.01}, main effects: threat, *b* =1.42, *SE* =.21, *t* =6.74, *p* <.001, CI {1.01;1.84}, norms country-level: *b* = -1.35, *SE* = .18, *t* =-.77, *p* =.44, CI {-.48;.21}.

*Autonomy-oriented helping*: *b* = -.16, *SE* = .08, *t* = -1.99, *p* = .05, CI {-.31;.00}, main effects: threat, *b* = -.21, *SE* =.31, t =-.70, *p* =.486, CI {-.82;.39}, norms on country-level: *b* =-.16, *SE* =.08, *t* =-2.00, *p* =.048, CI {-.31; -.00}

**Study 1B**

*Dependency-oriented helping*: *b* = .11, *SE* = .05, *t* = 2.24, *p* = .026, *CI* {.01; .21}, R^2^-change: .01, main effects: threat: *b* = -.71, *SE* =.24, *p* <.001, *CI* {-1.05; -.37}, norms country-level, *b* = -.20, *SE* = .05, *t* =-.96, *p* =.391, *CI* {-.65; .25}.

*Discrimination*: *b* = -.09, *SE* = .03, *t* = -2.82, *p* = .005, *CI* {-.16; -.03}, main effects: *b* = .93, *SE* =.11, *t* =8.44, *p* <.001, *CI* {-.05; .52}, norms country-level: *b* =.24, *SE* =.03, *t* =-2.82, *p* =.110, *CI* {-.05; .52}.

*Autonomy-oriented helping*: *b* = .03, *SE* = .04, *t* = 0.69, *p* = .49, *CI* {-.06; .12}, main effects: threat, *b* = -.65, *SE* =.15, *t* =-.4.41, *p* <.001, *CI* {-.94; -.36}, norms country-level, *b* = -.03, *SE* =.19, *t* =-.17, *p* =.864, *CI* {-.422; .354}.

**Controlling for autonomy-oriented helping in interaction of social norms and threat on dependency-oriented helping**

*Study 1A: b* = .28, *SE* = .05, *t* = 5.71, *p* <.001, CI {-.422; .354}. R^2^ change= .066.

*Study 1B*: *b* = .10, *SE* =.03, *t* =3.09, *p* =.002, CI {.04; .16}. R^2^ change= .011.

# Study 2A/B

**Results of social norms on perceived country/city level and threat on dependent measures.**

*Dependency-oriented helping*, *b* = .08, *SE* = .12, *t* = .66, *p* =.513 CI {-.16, .32}, R^2^-change: .00., main effects: threat, *b* =-.37, *SE*=.36, *t* =-1.03, *p* =.305, *CI* {-1.07; .33}, norms city-level, *b* =-.38, *SE* =.25, *t* =-1.49, *p* =.140, *CI* {-.88; .13}.

*Discrimination*, *b* =-.144, *SE* =.09, *t* =-1.68, *p* =.097, *CI* {-.32; .027}, main effects: threat, *b* =1.01, *SE* = .25, *t* =4.02, *p* <.001, *CI* {.51; 1.50}, norms city-level, *b* = .06, *SE* = .18, t=.36, *p* =.722, *CI* {-.29; .42}.

*Autonomy-oriented helping*: *b* =-.10, *SE* =.11, *t* =.-.86, *p* =.393, *CI* {-.31; .12}, main effects: threat, *b* = -.22, *SE* =.32, *t* =-.68, *p* =.497, *CI* {-.86; .42}, norms city-level: *b* =.27, *SE* =.23, *t* =1.17, *p* =.243, *CI* {-.19; .73}.

**Study 2B:**

*Dependency-oriented helping*: *b* =.37, *SE* =.27, *t* =.1.35, *p* =.178, *CI* {-.17; .90}, main effects: threat, *b* = -.99, *SE* =.91, *t* =-.1.09, *p* =.278, *CI* {-2.80; .81}, norms country-level: *b* =-.79, *SE* =.55, t=-1.45, *p* =.15, *CI* {-1.87; .29}.

*Discrimination*: *b* =-.38, *SE* =.17, *t* =-2.25, *p* =.026, *CI* {.711; -.05}, main effects: threat, *b* =2.02, *SE* =.57, *t* =3.55, *p* =.001, *CI* {.90; 3.15}, norms country-level, *b* = .44, *SE* =.34, *t* =1.30, *p* =.196, *CI* {-.23; 1.10}.

*Autonomy-oriented helping*: *b* = .38*, SE* =.19, *t* =1.98, *p* =.050, *CI* {.00; .75}, main effects: threat, *b* =-2.26, *SE* =.64, *t* =-3.52, *p* =.001, *CI* {-3.53; -.99}, norms country-level, *b* =-.70, *SE* =.39, *t* =-1.82, *p* =.072, *CI* {-1.46; .06}.

**Controlling for autonomy-oriented helping in interaction of social norms and threat on dependency-oriented helping**

*Study2A*: *b* = .28, *SE* =.07, *t* =3.88, *p* <.001, CI {,14; .42}. R^2^ change= .122

*Study 2B*: *b* = .17, *SE* =.12, *t* =1.49, *p* =.139, CI {-.06; .40}. R^2^ change=.017

# Study 3

**Results of social norms on perceived country and threat on dependent measures.**

*Dependency-oriented helping*: *b* =.08, *SE* =.05, *t* =1.46, *p* =.146, *CI* {-.03; .18}, main effects: threat, *b* =-.80, *SE* =.17, *t* =-4.79, *p* <.001, *CI* {-1.13; -.47}, norms country-level, *b* =.09, *SE* =.24, *t* =.35, *p* =.726, *CI* {-.39; .56}.

*Discrimination*: *b* = .04, *SE* =.05, *t* =.81, *p* =.419, *CI* {-.06; .13}, main effects: threat, *b* =.52, *SE* =.15, *t* =3.46, *p* =.001, *CI* {-.16; -.03}, *CI* {.23; .82}, norms country-level, *b* = -.45, *SE* =.22, *t* =-2.04, *p* =.042, *CI* {-.88; -.02}.

*Autonomy-oriented helping*: *b* =-.01, *SE* =.05, *t* =.27, *p* =.787, *CI* {-.12; .09}, main effects: threat, *b* = -.55, *SE* =.17, *t* =-.3.25, *p* =.001, *CI* {-.89; -.22}, norms country-level: *b* =.54, *SE* =.25, *t* =2.18, *p* =.030, *CI* {.05; 1.02}.

**Study 3 Results of charity-appeal dependent measure**

Participants have been told that we are interested in the perception of different online formats (written vs audio vs visual). Thus, they would see either a video, a short interview to listen to or an excerpt from a newspaper article. After, they were told to see two charity appeals (as a selection of 20 cases) in response to the consequences of a flooding (see similar Bareket et al., 2023). All participants received a newspaper article which was published on Jerusalempost.com (accessible [here](https://www.jpost.com/israel-news/article-692644)) translated to Hebrew with minor adaptations to account for accuracy (for example we indicated in the quote by a former member of the Israeli parliament that he does not occupy the position anymore by adding “former”). After reading the newspaper article, participants saw two charity appeals displayed on the same page in randomized order and asked how much of their potential lottery win they would distribute to the two charity appeals.

***Helping types by supporting charity-appeals.*** After reading the article about the consequences of the flooding, participants had the option to use their potential 100 NIS lottery win to support two charity appeals. Both appeals were stemming from Arab Israelis indicated by their names and presented in a randomized order. One appeal was describing the loss of one’s business and the other one the loss of one’s home due to the flooding. We conceptualized the intention to support the business as autonomy-oriented help and support for the loss of the home as dependency-oriented help. On mere numbers we did expect higher support for the charity-appeal with the lost home, but relevant for our purposes were mainly the values considering the interaction with norms and threat perceptions. Participants were asked to indicate the amount they would use to compensate for Mohammad (dependency-help), Ali (autonomy-help) and the amount to keep for themselves.

***Helping types by supporting charity-appeals.*** After reading the article about the consequences of the flooding, participants had the option to use their potential 100 NIS lottery win to support two charity appeals. Both appeals were stemming from Arab Israelis indicated by their names and presented in a randomized order. One appeal was describing the loss of one’s business and the other one the loss of one’s home due to the flooding. We conceptualized the intention to support the business as autonomy-oriented help and support for the loss of the home as dependency-oriented help. On mere numbers we did expect higher support for the charity-appeal with the lost home, but relevant for our purposes were mainly the values considering the interaction with norms and threat perceptions. Participants were asked to indicate the amount they would use to compensate for Mohammad (dependency-help), Ali (autonomy-help) and the amount to keep for themselves.

**Helping types by supporting charity appeals.**

People kept the largest amount to themselves (*M* = 54.83, *SD* = 40.19) followed by almost equal amounts they would distribute between Mohammed (dependency-oriented help, *M* = 22.94, *SD* =26.32) and Ali (autonomy-oriented help, *M* =22.23, *SD* =24.24).

There is no interaction between norms to support Arab Israelis on a country-level with threat perceptions on the amount participants offered for the dependency-helping charity appeal, *b* = -.23, *SE* = 1.29, *t* = -1.86, *p* = .064, 95% CI {-4.95, .14}, *R*^2^= .009, nor with norms in the social environment*, b* = -1.05, *SE* = .78, *t* = -1.36, *p* = .176, 95% CI {-2.58, .47}, *R*^2^=.005. Also, but as expected, there is no interaction on the amount participants offered for autonomy-oriented helping on country-level norms, *b* = -1.23, *SE* = 1.18, *t* = -1.04, *p* =.30, 95% CI {-3.55, 1.09}, *R*^2^= .003, nor with norms in the social environment, *b* = -.33, *SE* = .71, *t* = -.47, *p* =.638, 95% CI {-1.73, 1.06}, *R*^2^= .001. The amount of money that people kept to themselves also did not yield significant results, interaction of threat and norms on country-level, *b* = 3.63, *SE* = 1.92, *t* = 1.89, *p* =.06, 95% CI {-.14, 7.40}, *R*^2^= .009, norms in close social environment, *b* = 1.39, *SE* = 1.15, *t* = 1.20, *p* = .230, 95% CI {-.88, 3.66}, *R*^2^=.004. Consequently, using the charity appeal measurement did not yield support for our hypotheses. The limitation in the study design could explain the limited support for our hypotheses. Future research should realize a multi-factorial design in which also the social group of the person who is requesting help is systematically varied. In detail, manipulating if the charity appeal is coming from a Jewish Israeli vs Arab Israeli in dependency-oriented or autonomy-oriented ways could yield more thorough insights into the processes. In addition, the measurement differed from the measure in the previous study in this paper that the support was requested. Nadler et al. (2014) have found that when help recipients specifically request help, that this might impact the provision of the type of help. In more detail, they found that when disadvantaged group members specifically ask for autonomy-oriented help, this may increase the provision of it. Thus, even though no numerical differences were present, the charity appeal in which the disadvantaged group members asked for specific type of help might have impacted the levels of dependency-oriented and autonomy-oriented helping and consequently making it harder to find our proposed relationships.

**Results Study 2A Social Dominance Orientation**

We assumed that the provision of dependency-oriented help is also impacted by the motivation to maintain hierarchy. First evidence was shown by Halabi et al. (2008) that showed that Social Dominance Orientation (SDO, Pratto et al., 1994) – the tendency to see group hierarchies as legitimate – is related to the provision of dependency-oriented helping. In Study 2a we bring this together with social norms and show that rising levels of SDO go along with rising levels of dependency-oriented helping in combination with strong norms. That means, people who support social hierarchies may not only show discrimination (e.g. Michinov et al., 2005) or no help at all, but also helping behavior – in particular dependency-oriented helping behavior when they perceive strong norms to help.

***SDO.*** Social Dominance Orientation was measured using the short four-item scale from Pratto et al. (2013), for example, “We should not push for group equality (rev.)”, *α* = .79. As expected, entering SDO instead of threat perceptions into the interaction model yielded a significant interaction, *b* = .21, *SE* = .09, *t* = 2.32, *p* = .023, CI {.03, .39}, *R*^2^= .05.^[[1]](#footnote-1)^

# Experimental Study S1

The aim of this study was to test in an experiment whether induced threat and perceived norms to help refugees interact, predicting the tendency to show dependency-oriented helping and discrimination (but not autonomy-oriented helping). We induced threat in the experimental condition and measured the extent to which norms to help refugees were perceived by participants as central in their social environment. We focused on perceived economic competition to induce realistic threat as past research has shown a relationship between perceived realistic threat (e.g. intergroup economic competition) and forms of dependency-oriented helping and discrimination (Esses et al., 1998, 2001; Jackson & Esses, 2000; Stephan et al., 2009). Thus, we tested the following hypotheses based on expected interactions of experimental condition with perceived norms:

H1: Participants in the threat condition, as compared to a control condition, show a higher intention to provide dependency-oriented help when they perceive strong (vs. weak) norms to help refugees. No such effect should be found for autonomy-oriented help.

H2: Participants in the threat condition will be less likely to engage in overt discrimination when they perceive strong norms to help refugees, and more likely to engage in overt discrimination when they perceive no, or weak norms to help refugees.

## Method

**Design and sample**.^[[2]](#footnote-2)^ The reported experiment was a one-factor design with two levels (threat manipulation, threat control), a continuous moderator variable (perceived social norms to help refugees), and two dependent variables (dependency-oriented helping and discrimination). Participants were randomly assigned to either the experimental or the control condition. The experiment was conducted online via snowball sampling. We excluded five participants who indicated that they did not read the manipulation stimuli, resulting in a total sample of *N* = 110. Participant age ranged from 18-66 years (*M* = 28.49, *SD* = 10.88). The sample comprised 28% males, 71% females, and 1% intersex with a mixed educational background, with 43% of them with an academic background (among them also students).

#### Materials

**Threat manipulation.** To manipulate threat, we developed two vignettes. In order to induce economic threat, we stated that refugees are in general very well-educated and have a promising future regarding their occupational career. We included parts of a German newspaper article, discussing the expansion of the institution that deals with accreditation and recognition of refugees’ professional qualifications. The vignette also informed that preferential treatment of Germans and Europeans on the job market (which was policy at the time) will be officially dropped. We also included in the vignette that a number of spots in coveted university seminars would be specifically reserved for refugees, thus reducing the spots available for German students. This part of the vignette included quotes from a student saying the he feared not being able to finish his studies on time because of this.

In the control condition, participants read an article which described refugees as having mixed educational backgrounds. The article included reference to administrative obstacles that refugees may encounter in the job market. Regarding university entrance, it was stated that refugees do not receive special treatment and actually have more difficulties because they often lack the necessary papers and certificates to enroll in university courses.

**Credibility.** With two items, we asked participants if they thought the vignettes were reliable and trustworthy (1 = completely disagree, 7 = completely agree), α = .90.

**Manipulation check.** We asked participants how they perceived the opportunities for refugees on the job market (1 = very low opportunities, 7 = very high opportunities). We also asked them how they thought the increase in refugees might affect overall job availability (1 = Strongly reduces job availability, 7 = Strongly increases job availability), and if German students’ access to university seminars would be limited (1 = completely disagree, 7 = completely agree), α = .69.

**Helping intentions.** To measure helping intentions we used an eight-item scale developed by Becker et al. (2017), measuring dependency and autonomy-oriented helping with four items respectively. Dependency-oriented helping was for example measured with “I would support an initiative that distributes food and beverages in front of a refugee shelter”. In contrast, an example for autonomy-oriented helping is “I would support an initiative that informs refugees about their rights in Germany” (α_dependency_ = .76, α_autonomy_ = .74; 1 = completely disagree, 7 = completely agree). We included autonomy-oriented helping for validity reasons: Only the specific form of dependency-oriented helping should be affected by an interaction of norms and threat.

**Discrimination***.* The scale included items such as, “Our borders should be secured better” and “I don’t support any policy measures that try to help refugees” (α = .87, 1 = completely disagree 7 = completely agree, full list in online supplement).

**Perceived norms in social environment.** We used only norms in close social environment with seven-item scale (similar to Study 1-3) to gauge the extent to which participants perceived norms to help refugees in their social environment (e.g. friends, family members). The scale included items such as, “People in my social environment think there should be more assistance for refugees” (α = .86, 1 = completely disagree 7 = completely agree, full list in online supplement).

**Procedure**

Participants were invited to take part in an online survey on decision making and actions. The invitation included formal information about the study and data privacy. After consenting to participate, participants were randomized to first read either the newspaper article crafted to induce threat or the control article. They were then asked questions about article credibility and given the manipulation check. After reading a second newspaper article and answering further questions (see Footnote 1), participants responded to the scales regarding reactions towards refugees (intention to provide dependency/ autonomy-oriented help and avoidant-discrimination). Participants were asked about their agreement with paternalism as well as the perceived norms in their social environment to help refugees. Finally, we tapped participant demographic information, after which participants were debriefed.

## Results

**Pre-analyses**. Credibility rating resulted in *M* = 4.72 (*SD* = 1.46) and the vignettes of the threat manipulation did not differ significantly regarding their credibility, *M*_threat_ = 4.96 (SD = 1.40), *M*_control_ = 4.47 (*SD* = 1.50), *t*(108) = –1.75, *p* = .083. The manipulation check revealed a successful manipulation of threat perception: *M*_threat_ = 2.91 (*SD* = 1.03), *M*_control_ = 2.05 (*SD* = .89), *t*(108) = – 4.68, *p* < .001, *d* = 0.89. People felt significantly more economically threatened by refugees in the threat condition compared to the control condition. Table 1 shows means, standard deviations, and correlations of all variables measured.

*Table SM2.*

Correlations among Main Variables in the Experimental Study.

|  | *M* | *SD* | 1. | 2. | 3. |
| --- | --- | --- | --- | --- | --- |
| 1. Norms social environment | 4.69 | 1.26 |  |  |  |
| 1. Autonomy-oriented-help | 4.56 | 1.40 | .58** |  |  |
| 1. Avoidant-discrimination | 2.33 | 1.14 | -.65** | -.61** |  |
| 1. Dependency-oriented help | 4.82 | 1.40 | .40** | .65** | -.56** |

*Note.* ***p* < .01, scales range from 1-7

**H1: Moderation analysis for dependency-oriented helping.** Testing whether the effects of perceived threat on dependency-oriented help intentions were moderated by perceived norms to help refugees, the same predictor variables as above were entered in a moderated regression analysis. Again, the overall model was significant: *F*(3,106) = 8.25, *p* < .001, *R^2^* = .19. The analysis showed no significant overall interaction of threat and norms on dependency-oriented help intentions, *b* = .28, *SE* = 0.20, *t*(106) = 1.42, *p* = .16, Δ*R^2^* = .015. However, the relationship was in the hypothesized direction (see Figure 1, intermediate panel). Participants in the threat condition, as compared to the control condition, were more likely to demonstrate dependency-oriented helping intentions if they perceived strong norms to help refugees (+1 *SD*), *b* = 0.70, *SE* = 0.35, *t*(106) = 2.02, *p* = .045, CI 95% [0.2;1.39], but not when they were medium or low (-1 *SD*), *b* = .00, *SE* = 0.35, *t*(106) = 0.01, *p* = .995. Comparable analyses for autonomy-oriented help yielded only a significant main effect of norms, but there was no detectable influence of threat perception on autonomy-oriented help intentions at any level of perceived norms, *b* = .28, *t*(106) = 0.91, *p* = .367. This suggests that the impact of threat was specific to dependency-oriented help, as expected.

**H2: Moderation analysis for discrimination**. Testing whether the effect of perceived threat on discrimination was moderated by perceived norms to help refugees, we found that the overall model was significant, *F*(3,105) = 29.10, *p* < .001, *R*^2^ = .45. Again, the analysis revealed that perceived norms moderated the effect of threat on discrimination, *b* = -.35, *SE* = 0.13, *t*(106) = -2.65, *p* = .009, Δ*R^2^=. 037*. The lower panel C of Figure 1 shows that when norms were weak (-1 *SD*), discrimination against refugees was higher in the threat condition than in the control condition (*M*_control_ = 2.75, *M*_threat_ = 3.26, *b* = .50, *SE* = .24, *t* = 2.11, *p* = .037, CI 95% [.03; .97]). In contrast, when norms to help refugees were perceived to be strong (+1 SD) this relationship was rendered non-significant and changed direction (*M*_control_ = 1.78, *M*_threat_ = 1.39, *b* = -.38, *SE* = .23, *t* = -1.65, *p* =.102, CI 95% [-.85; .08]).


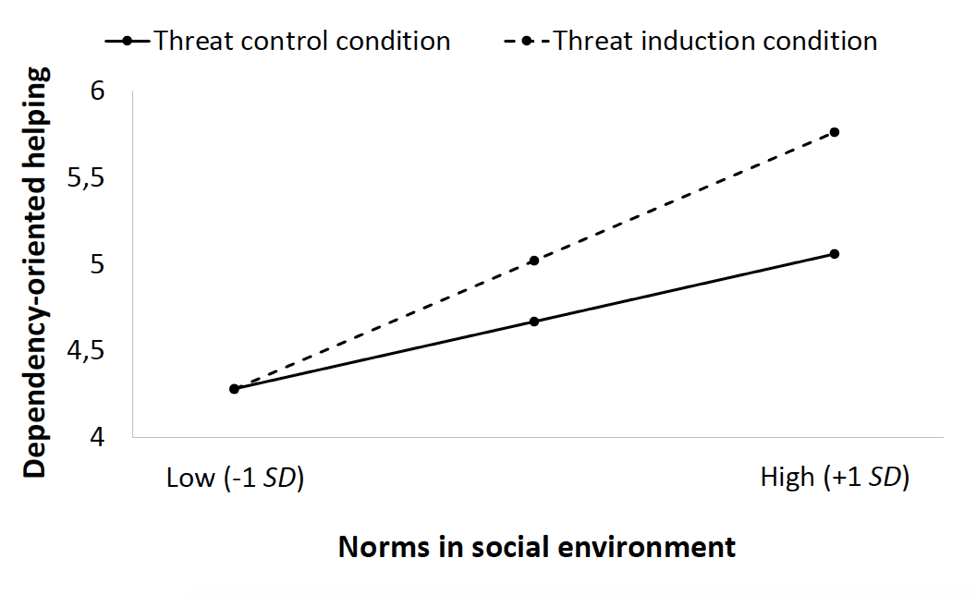


B

A


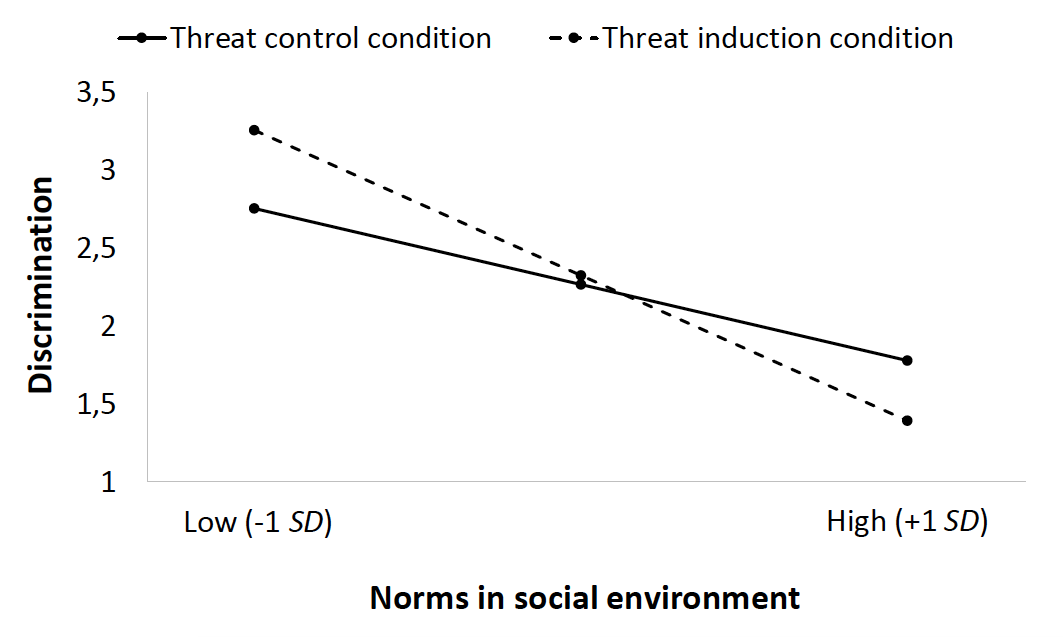


*Figure 1.* Conditional effects of perceived norms and threat on dependency-oriented helping (A) and discrimination (B).

**Results of specific threat**

**Study 1A Germany refugees**

All subtypes of threat – realistic threat, symbolic threat, safety threat – show the same pattern on dependency-oriented helping.

Realistic threat interaction with social norms: *b*= .23, *SE*=.05, *t*=4.32, *p*<.001

Symbolic threat interaction with social norms: *b*= .22, *SE*= .05, *t*= 4.15, *p*<.001

Safety threat interaction with social norms: *b*= .22, *SE*=.05, *t*=4.44, *p*<.001

**Study 1B Israel refugees**

Subtypes realistic threat and safety threat showed significant interaction with social norms.

Realistic threat: *b*= .10, *SE*=.04, *t*=2.35, *p*=.019

Safety threat: *b*=.09, *SE*=.04, *t*=2.17, *p*=.030

Symbolic threat shows similar pattern but not significant interaction: *b*=.06, *SE*=.04, *t*=1.57, *p*=.118

**Study 2A Germany refugees**

All subtypes show significant interaction with social norms.

Realistic threat: *b*=.27, *SE*= .08, *t*=3.55, *p*<.001

Symbolic threat: *b*= .21, *SE*=.06, *t*=3.68, *p*<.001

Safety threat: *b*=.27, *SE*=.07, *t*=4.04, *p*<.001

**Study 2B Germany Ukrainian refugees**

Only safety threat shows significant interaction with social norms.

Safety threat: *b*=.30, *SE*=.12, *t*=2.53, *p*=.013

Realistic threat: *b*=.05, *SE*=.08, *t*=.60, *p*=.551

Symbolic threat: *b*=.10, *SE*=.12, *t*=.91, *p*=.367

**Study 3 Israel Arab Israelis**

Subtypes symbolic threat and safety threat show significant interactions with social norms.

Symbolic threat: *b*= .07, *SE*=.03, *t*= 2.27, *p*=.024

Safety threat: *b*= .08, *SE*=.03, *t*=2.56, *p*=.011

Realistic threat shows same direction but does not reach significance, *b*= .04, *SE*= .03, *t*=1.18, *p*= .239

# References OS

Bareket, O., Ein-Gar, D., & Kogut, T. (2023). I will help you survive but not thrive: Helping decisions in situations that empower women. *Group Processes & Intergroup Relations*, *26*(7), 1641–1659. https://doi.org/10.1177/13684302221108437

Becker, J. C., Ksenofontov, I., Benz, A., & Bogert, L. (2017). Einstellungen und Verhaltensweisen gegenüber Geflüchteten. In A. Rohmann & S. Stürmer (Eds.), *Die Flüchtlingsdebatte in Deutschland – Sozialpsychologische Perspektiven. Beiträge zur Angewandten Psychologie*. Peter Lang.

Esses, V. M., Dovidio, J. F., Jackson, L. M., & Armstrong, T. L. (2001). The immigration dilemma: The role of perceived group competition, ethnic prejudice, and national identity. *Journal of Social Issues*, *57*, 389–412. https://doi.org/10.1111/0022-4537.00220

Esses, V. M., Jackson, L. M., & Armstrong, T. L. (1998). Intergroup competition and attitudes toward immigrants and immigration: An instrumental model of group conflict. *Journal of Social Issues*, *54*, 699–715. https://doi.org/10.1111/j.1540-4560.1998.tb01244.x

Halabi, S., Dovidio, J. F., & Nadler, A. (2008). When and How Do High Status Group Members Offer Help: Effects of Social Dominance Orientation and Status Threat. *Political Psychology*, *29*(6), 841–858. https://doi.org/10.1111/j.1467-9221.2008.00669.x

Jackson, L. M., & Esses, V. M. (2000). Effects of perceived economic competition on people’s willingness to help empower immigrants. *Group Processes & Intergroup Relations*, *3*, 419–435. https://doi.org/10.1177/1368430200003004006

Michinov, N., Dambrun, M., Guimond, S., & Méot, A. (2005). Social dominance orientation, prejudice, and discrimination: A new computer-based method for studying discriminatory behaviors. *Behavior Research Methods*, *37*(1), 91–98. https://doi.org/10.3758/bf03206402

Pratto, F., Çidam, A., Stewart, A. L., Zeineddine, F. B., Aranda, M., Aiello, A., Chryssochoou, X., Cichocka, A., Cohrs, J. C., Durrheim, K., Eicher, V., Foels, R., Górska, P., Lee, I.-C., Licata, L., Liu, J. H., Li, L., Meyer, I., Morselli, D., … Henkel, K. E. (2013). Social Dominance in Context and in Individuals: Contextual Moderation of Robust Effects of Social Dominance Orientation in 15 Languages and 20 Countries. *Social Psychological and Personality Science*, *4*(5), 587–599. https://doi.org/10.1177/1948550612473663

Pratto, F., Sidanius, J., Stallworth, L. M., & Malle, B. F. (1994). Social dominance orientation: A personality variable predicting social and political attitudes. *Journal of Personality and Social Psychology*, *67*(4), 741–763. https://doi.org/10.1037/0022-3514.67.4.741

Stephan, W. G., Ybarra, O., & Morrison, K. R. (2009). Intergroup threat theory. In *Handbook of prejudice, stereotyping, and discrimination* (pp. 43–59). Psychology Press. https://doi.org/10.4324/9781841697772

1. With dependency helping at measurement point 2 as SDO was only measured at week 4 . Controlling for dependency-oriented helping at measurement point 1 replicates the results: *b* =.20, *SE* =.08, *t* =2.51, *p* =.014, CI {.04, .35}, *R*^2^=.05. [↑](#footnote-ref-1)
2. The experiment was originally intended to be a 2 (threat, no threat) × 2 (norms, control) design. We checked if our norms manipulation was successful with five items (e.g. “People should help refugees” or “People and organizations that help refugees should be awarded”), scale ranging from 1-7 (1 = do not agree at all, 7 = do fully agree), α = .80. The manipulation check revealed that the norm manipulation did not work, *M*_norm_ = 5.13, *SD*_norm_ = 0.83, *M*_control_ = 5.22, *SD*_control_ = 0.93, *t*(218) = 0.71, *p* = .48. Thus, we do not report data from the conditions in which we intended to manipulate norms as we cannot be sure if the manipulation had other effects. We only report data from the conditions in which participants read the norms control vignette. Instead of the manipulation, we included perceived norms in the social environment as a potential moderator to test whether norms influence hierarchy-maintaining actions towards refugees. The result pattern does not change when all data are included in the analyses, though Δ*R^2^* is higher when only the control conditions are analyzed. [↑](#footnote-ref-2)
